# Psychometric Evaluation of the PRO-CTCAE Average Composite Score: Reliability, Responsiveness, Known-Groups Validity, and Sensitivity to Group Differences

**DOI:** 10.3390/cancers18142265

**Published:** 2026-07-15

**Authors:** Minji K. Lee, Amylou C. Dueck, Blake T. Langlais, Gina L. Mazza, Gita Thanarajasingam, Allison M. Deal, Brie N. Noble, Lauren Rogak, Tito R. Mendoza, Sandra A. Mitchell, Ethan Basch

**Affiliations:** 1 Department of Quantitative Health Sciences, Mayo Clinic, Rochester, MN 55905, USA; 2 Department of Quantitative Health Sciences, Mayo Clinic, Scottsdale, AZ 85259, USA; 3 Division of Hematology, Mayo Clinic, Rochester, MN 55905, USA; 4 Lineberger Comprehensive Cancer Center, University of North Carolina, Chapel Hill, NC 27599, USA; 5 Center for Cancer Research, National Cancer Institute, Rockville, MD 20850, USA; 6 Division of Cancer Control and Population Sciences, National Cancer Institute, Rockville, MD 20850, USA

**Keywords:** PRO-CTCAE Average Composite Score, test–retest reliability, responsiveness, known-groups validity, sensitivity to group differences, clinical trials

## Abstract

**Background/Objectives:** The PRO-CTCAE Average Composite Score (ACS), calculated as the mean of PRO-CTCAE composite scores, summarizes overall symptom and side effect burden. This study evaluated the test–retest reliability, responsiveness to change, known-groups validity, and sensitivity to group differences in the ACS. **Methods:** Analyses used the original PRO-CTCAE validation dataset, including lung (*n* = 174), breast (*n* = 222), and head and neck (*n* = 139) cancer cohorts, and data from the COMET-2 randomized phase III trial comparing cabozantinib (*n* = 53) and mitoxantrone–prednisone (*n* = 54) in men with previously treated prostate cancer. Test–retest reliability was assessed using intraclass correlation coefficients (ICCs) from two-way mixed-effects models for agreement. Responsiveness was evaluated by relating ACS change scores (Visit 1 minus follow-up Visit 2) to patient-reported global ratings of change (GRC; worsened, unchanged, or improved) using standardized response means (SRMs) and Jonckheere–Terpstra trend tests. Known-groups validity was examined by comparing mean ACS values between ECOG performance status groups (0–1 vs. 2–4). Sensitivity to treatment group differences was assessed using model-based area-under-the-curve (AUC) comparisons of longitudinal ACS trajectories. **Results:** Test–retest reliability was acceptable, with ICCs among GRC-defined stable patients of 0.80 (95% CI: 0.70–0.87) in lung cancer, 0.84 (95% CI: 0.77–0.89) in breast cancer, and 0.77 (95% CI: 0.63–0.86) in head and neck cancer; ICCs based on assessments completed one day apart ranged from 0.88 to 0.90. The ACS demonstrated responsiveness, with SRMs of 0.30/0.15/−0.37 (lung), 0.29/0.14/−0.40 (breast), and −0.01/−0.20/−0.56 (head/neck) for improved/no-change/worsened groups, respectively, and significant monotonic trends across GRC categories (all *p <* 0.01). Known-groups validity was supported by conceptually expected differences in ACS values across distinct levels of self-reported patient-reported physical functioning and ECOG performance status categories. Mean ACS values were lower among patients with good versus limited physical functioning (0.71 vs. 1.23 in lung cancer, 0.52 vs. 1.19 in breast cancer, and 0.69 vs. 1.29 in head and neck cancer) and among patients with ECOG PS 0–1 versus 2–4 (0.93 vs. 1.31, 0.74 vs. 1.22, and 0.90 vs. 1.06, respectively). In COMET-2, higher symptom burden was detected in the cabozantinib arm compared with the mitoxantrone–prednisone arm (AUC difference = 1.5, 95% CI: 0.2–2.8, *p* = 0.02). **Conclusions:** The ACS demonstrated acceptable test–retest reliability, responsiveness, and known-groups validity across multiple cancer populations. These findings support its use as a complementary summary measure of overall symptomatic adverse event burden alongside individual symptom-level analyses.

## Simple Summary

This study builds on our prior psychometric validation of the Patient-Reported Outcome version of the Common Terminology Criteria for Adverse Events (PRO-CTCAE^®^) Average Composite Score (ACS), published in this issue of *Cancers*. Although the earlier study focused on structural validity, including internal consistency, dimensionality, and factor model fit, the present work provides additional evidence supporting the use of the ACS in longitudinal and comparative research, using the same cancer cohorts. We evaluated the stability of ACS values when no change was expected (test–retest reliability), responsiveness to patient-reported improvement or worsening, discrimination between clinically distinct groups defined by self-reported physical function and ECOG performance status, and sensitivity to detecting treatment-related differences in randomized trial settings. Across multiple cancer populations, ACS changes aligned with patient-reported global ratings of change, and clinically distinct groups demonstrated appropriately different symptom burden. Extensive sensitivity analyses showed that these findings were robust across varying numbers and compositions of symptom terms, with greater stability achieved as more clinically relevant symptoms were included.

## 1. Introduction

Patient-reported outcomes (PROs) play a central role in evaluating symptomatic adverse events (AEs) in oncology clinical trials, providing critical information that complements clinician-reported toxicity grading. The U.S. National Cancer Institute’s Patient-Reported Outcomes version of the Common Terminology Criteria for Adverse Events (PRO-CTCAE) was developed to systematically capture symptom frequency, severity, and interference directly from patients and has demonstrated strong content validity, construct validity, reliability, and responsiveness at the item level across a wide range of cancer populations and treatments [1,2]. Consistent with its role as a complement to clinician-reported CTCAE, PRO-CTCAE data are commonly summarized at the item level or symptom level, either by analyzing AE-level symptom scores individually or by deriving symptom-specific endpoints, such as maximum post-baseline scores, baseline-adjusted symptom scores, or the proportion of patients exceeding clinically meaningful symptom thresholds (e.g., a maximum post-baseline score ≥3 for a given symptom) [3]. However, these approaches do not directly yield a single interpretable measure of overall symptom or side-effect burden across multiple concurrent symptoms, which remains challenging in trials evaluating cumulative toxicity [4].

To address this gap, we previously developed a PRO-CTCAE Average Composite Score (ACS) intended to summarize overall symptomatic adverse event burden across multiple AE-level composite scores [5]. In that work, we demonstrated strong evidence of structural validity of the ACS through confirmatory factor analyses, internal consistency estimates, and correlations with an established summary measure of health-related quality of life (the EORTC QLQ-C30 summary score), supporting interpretation of the ACS as a coherent latent construct within lung, breast, and head and neck cancer populations.

The conceptualization of multi-symptom summary measures such as the ACS raises important measurement model considerations. In particular, debate exists [6] as to whether such constructs are best represented using reflective models, in which a latent symptom burden construct gives rise to correlated symptom indicators, or formative (causal indicator) models, in which individual symptoms collectively define the construct. In prior work, evidence from internal consistency and factor-analytic models supported a reflective interpretation of the ACS. However, given the heterogeneous and context-dependent nature of symptomatic AEs, formative interpretations may also be highly relevant. Moreover, our prior findings suggested that the ACS and individual symptom-level analyses serve complementary roles, as symptom-level analyses retain more detailed multidimensional information and can reveal distinct symptom profiles among patients with similar ACS values.

This study is guided by the COnsensus-based Standards for the Selection of Health Measurement Instruments (COSMIN) frameworks [7,8,9], which provides a comprehensive taxonomy for evaluating the measurement properties of PRO instruments. Within this framework, evaluation extends beyond structural validity to include reliability between repeated measurements, responsiveness, and validity in relation to external criteria. For the ACS to summarize overall symptom burden and support longitudinal and comparative oncology research, additional psychometric evaluation is required. Accordingly, we prespecified the following hypotheses: (1) ACS values would demonstrate stability among patients whose symptom status is not expected to change supporting test–retest reliability, (2) ACS would show ordered separation across patient-reported global rating of change supporting responsiveness to change, (3) patients with worse patient- or physician-reported physical function status would report higher ACS values supporting known-groups validity, and (4) longitudinal ACS trajectories would differentiate treatment groups with distinct symptomatic AE profiles supporting sensitivity to group differences.

Regulatory guidance further emphasizes the importance of fit-for-purpose validation of PRO scores intended for use in cancer clinical trials. Recent U.S. Food and Drug Administration guidance on core patient-reported outcomes in oncology highlights summary measures capturing overall side-effect impact as core endpoints in addition to symptomatic AEs and physical function and specifies that their use should be supported by robust psychometric evidence appropriate to the intended application [10]. Together, these methodological and regulatory considerations motivate a comprehensive evaluation of the PRO-CTCAE ACS beyond structural validity alone.

In this study, we extend prior work by evaluating additional measurement properties of the PRO-CTCAE ACS. Using data from the original PRO-CTCAE validation study and the COMET-2 randomized trial, this work provides a comprehensive assessment of the ACS as a parsimonious and interpretable summary measure of overall symptomatic AE burden in oncology research.

## 2. Materials and Methods

### 2.1. Data Sources

This study included secondary analyses of two data sources. The first was the multi-site PRO-CTCAE validation dataset (clinicaltrials.gov: NCT02158637), which was used to evaluate psychometric properties of PRO-CTCAE measures [1], including the structural validity of the ACS reported previously in this Special Issue [5]. The same lung, breast, and head and neck cancer cohorts included in the prior structural validity analyses were used in the present study to evaluate test–retest reliability, responsiveness, and known-groups validity of the ACS. These cohorts were selected because they provided sufficient sample sizes for the psychometric analyses while also enabling extension of the previously reported structural validity findings.

The second data source was the COMET-2 randomized clinical trial (clinicaltrials.gov: NCT01522443) [11], which has supported prior PRO-CTCAE-focused methodological work [12,13,14]. The COMET-2 compared cabozantinib s-maleate (60 mg once daily) with mitoxantrone hydrochloride (12 mg/m^2^ every 3 weeks) plus oral prednisone (5 mg twice daily) in 107 men with metastatic castration-resistant prostate cancer randomized between 2012 and 2014. The trial was terminated early following results from the companion COMET-1 trial, which demonstrated no survival benefit for cabozantinib [15]. In COMET-2, cabozantinib did not improve the primary endpoint of pain palliation compared with mitoxantrone–prednisone. However, prior analyses of PRO-CTCAE data demonstrated notable between-arm differences in the symptomatic AE profile, with patients receiving cabozantinib reporting higher frequencies of several non-pain symptoms including decreased appetite, diarrhea, and nausea [12,13]. Treatment differences were not uniform across symptom domains; some symptoms seemed to favor mitoxantrone, whereas others showed little difference between treatments. Consequently, there was no clear overall superiority of either treatment with respect to total symptom burden when considering individual AEs in isolation. This pattern of heterogeneous, multidimensional symptom profiles made COMET-2 an informative setting for evaluating whether the ACS could summarize cumulative symptomatic AE burden and detect between-group differences in a randomized trial context.

### 2.2. Study Samples

**Figure 1:**
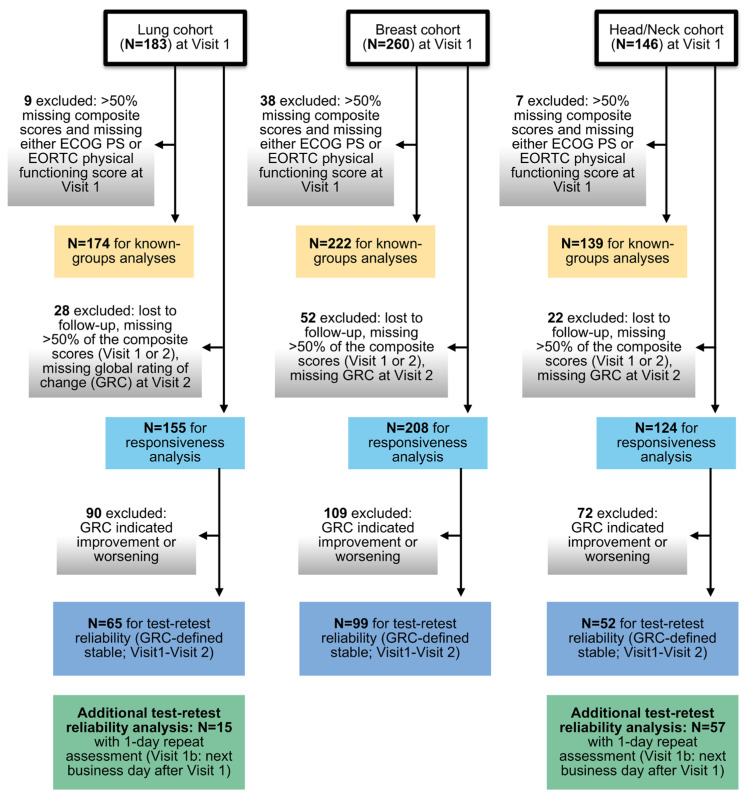
CONSORT diagram for test–retest reliability, responsiveness, and known-groups analyses. *Note.* GRC = Global Rating of Change; Visit 2 data were collected between 1 and 6 weeks after Visit 1. The full Visit 1 cohort (*n* = 183 in lung cancer, *n* = 260 in breast cancer, and *n* = 146 in head and neck cancer) was previously used in the initial ACS validation study [5] to evaluate structural validity, whereas the current study uses subsets of that cohort for additional psychometric evaluations.

The samples used for test–retest reliability, responsiveness and known-groups validity analyses are described in the CONSORT diagram (Figure 1). Known-groups validity analyses were conducted using Visit 1 measurements. Responsiveness analyses were based on data from Visit 1 and Visit 2 (1 to 6 weeks apart). Test–retest reliability was evaluated using two approaches: (1) Visit 1 and Visit 2 data (1 to 6 weeks apart), and (2) a subset of participants with repeat assessments one day apart (Visit 1 and Visit 1b). Between-arm differences in ACS trajectories were evaluated in the 107 COMET-2 patients. Sample characteristics for the PRO-CTCAE validation study and COMET-2 trial have been reported previously [1,5,11,12].

### 2.3. Symptom Terms and ACS Calculation

PRO-CTCAE allows investigators to select disease- and treatment-relevant symptom terms rather than mandating a fixed symptom set. While this flexibility supports contextual relevance, it also introduces variability in measurement choices across studies. To align analyses with prior work and to ground symptom selection in established content-validity work, we used disease-specific symptom sets that had previously been identified through patient- and clinician-informed methodologies, including qualitative interviews, symptom prevalence assessments, relevance and importance ratings, and consensus-based selection processes [16,17,18,19,20,21,22,23,24]. These studies identified symptom terms that were considered particularly relevant, prevalent, and/or important for patients within specific disease settings and provided the foundation for symptom selection in the present analysis. Using the symptom terms available within the PRO-CTCAE validation dataset, we examined eight symptom terms for lung cancer [24], 16 terms for breast cancer [20], and 17 terms for head and neck cancer [22]. The specific symptom terms for each cohort are provided in Supplementary Materials: Table S1 and have also been reported previously [5].

PRO-CTCAE measurement is based on items measuring frequency, severity, interference, amount, or presence, with each symptomatic AE represented by one to three item responses. To provide a single representative score per symptom term, we applied the PRO-CTCAE composite scoring algorithm [25] developed through National Clinical Trials Network cooperative group consensus procedures. This algorithm transforms combinations of item responses from the original 0–4 ordinal scales into a composite score ranging from 0 to 3. Composite scores were generated using the ‘toxScores’ function in the R package, ‘ProAE’ (version 1.0.3) [26].

The ACS was calculated for each participant as the mean of the available AE-level composite scores. Because the ACS is defined as the average of AE-level composite scores, and each composite score ranges from 0 to 3, the ACS therefore remains on a consistent 0–3 scale regardless of the number of symptom terms included. Participants were required to have non-missing data for at least 50% of the symptom composites contributing to the ACS. This 50% non-missingness threshold was informed by prior simulation results demonstrating that person-mean imputation performs comparably to more complex methods, such as random forest imputation, in terms of mean absolute error and root mean squared error when the proportion of missing data is ≤50%, supporting its use within this range [27].

Patterns of data completeness varied across disease groups due partly to missing-by-design questionnaire administration in the original validation study. In the lung cohort, the majority of participants (90–91%) had complete data on all 8 composites across timepoints. In the breast cohort, 49–50% of participants had complete data on all 16 symptom composites, while an additional 42% had data on 12 composites at both timepoints (75% completion). In the head and neck cohort, 65–66% of participants had complete data on all 17 symptom composites, and 27–29% had data on 12 composites (70% completion). We applied this ≥50% non-missing response criterion consistently across all primary and sensitivity analyses. This yielded analytic sample sizes of 174 for lung, 222 for breast, and 139 for head and neck cancer, each contributing to at least one analysis in this study. In the COMET-2 trial, ACS was computed using 10 symptomatic AEs (insomnia, constipation, pain, fatigue, nausea, vomiting, diarrhea, decreased appetite, numbness/tingling, and shortness of breath) at each of the six timepoints (baseline, month 1, month 2, month 3, month 6, and month 8).

### 2.4. Statistical Analyses

#### 2.4.1. Test–Retest Reliability of the ACS

Test–retest reliability was evaluated using two complementary definitions of stability: (1) participants who completed repeat assessments one day apart (Visit 1b, administered one day after Visit 1), a short interval during which minimal true change in symptom experience is expected given the 7-day recall period of the PRO-CTCAE items, and (2) participants reporting that their physical condition remained “about the same” on the global rating of change (GRC) item between Visit 1 and Visit 2 (1–6 weeks apart). Test–retest reliability was quantified using intraclass correlation coefficients (ICCs) based on a two-way random-effects model for single measurements and absolute agreement (ICC(A,1)) [28]. Primary analyses used all disease-relevant symptom terms available for each cancer cohort.

To assess robustness of test–retest reliability to symptom selection, we conducted an exact combinatorial subset sensitivity analysis, enumerating all possible subsets of size *k* ≥ 3. For GRC-defined test–retest reliability sensitivity analyses, the subset sizes were *k* = 3–8 for lung cancer, *k* = 3–16 for breast cancer, and *k* = 3–17 for head and neck cancer.

A subset of participants completed repeat assessments one day apart (Visit 1b), among those undergoing daily radiation or chemoradiation therapy. In this subsample, PRO-CTCAE items corresponding to 26 symptomatic AEs were administered, yielding 5 symptom terms relevant for lung cancer and 11 for head and neck cancer. Among those participants, 15 in the lung cohort and 57 in the head and neck cohort met the >50% non-missing response criterion to compute the ACS. No breast cancer patients had Visit 1b assessments. Accordingly, for Visit 1b-based test–retest reliability sensitivity analyses, subset sizes ranged from *k* = 3–5 for lung cancer and *k* = 3–11 for head and neck cancer. For each subset, ACS was calculated, and ICCs were estimated. The distribution of ICC values across all subsets was summarized using the median, interquartile range (IQR), and range. An ICC value ≥ 0.70 [29] was deemed acceptable.

#### 2.4.2. Responsiveness

Responsiveness was evaluated by computing ACS change scores between Visit 1 and Visit 2 and relating them to patients’ global rating of change (GRC) in physical condition, categorized as worsened, unchanged, or improved. Change scores were calculated as Visit 1 minus Visit 2 ACS (Visit 1 − Visit 2), such that positive values indicated symptom improvement. Responsiveness was summarized using standardized response means (SRMs), calculated as the mean change divided by the standard deviation of the change scores within each GRC group. Jonckheere–Terpstra trend tests were used to assess whether ACS change scores demonstrated a monotonic trend across ordered GRC categories.

Because PRO-CTCAE does not mandate a fixed set of symptom terms for a given disease group, sensitivity analyses were conducted to evaluate the robustness of responsiveness to symptom term selection. For each disease cohort and each subset size ranging from *k* = 3 to the total number of terms, all possible symptom subsets were enumerated. For each subset, ACS was recomputed at Visit 1 and Visit 2. SRMs were estimated separately within each GRC category. For each subset size, the distribution of SRMs across all enumerated symptom subsets was summarized using the median, IQR, and range.

#### 2.4.3. Known-Groups Validity

Known-groups validity was evaluated by comparing the mean baseline (Visit 1) ACS values between good versus poor patient-reported physical functioning based on the EORTC QLQ-C30 scores (good physical function ≥ 83 versus poor physical function < 83 [30]). For the primary analyses, mean ACS values were compared between groups using two-sample *t*-tests, and the magnitude of the between-group differences was quantified using Cohen’s *d* effect sizes. We hypothesized that patients with good physical functioning would report lower symptom burden (i.e., lower ACS values) than patients with poor physical functioning. These analyses were repeated using physician-reported Eastern Cooperative Oncology Group performance status (ECOG PS) as a complementary known-groups anchor (ECOG PS 0–1 vs. ECOG PS 2–4). Correlations between the ACS and each grouping indicator were also evaluated.

To assess the robustness of known-groups validity to the number and composition of symptom terms included in the ACS, sensitivity analyses were conducted using a complete subset enumeration approach. For each disease cohort and each subset size ranging from *k* = 3 to the total number of disease-specific symptom terms, all possible symptom subsets were enumerated. For each subset, the ACS was recalculated as the mean of the selected symptom-term composite scores. Mean ACS values for each known-groups category (good vs. poor physical functioning and, separately, ECOG 0–1 vs. ECOG 2–4) and the difference in means between groups were recorded for each subset. These differences were used to evaluate both the magnitude and direction of known-groups separation across symptom subsets. For each subset size (*k*), the distribution of group differences across all enumerated subsets was summarized using the median, IQR, and range. In addition, the proportion of subsets exhibiting the expected ordering (e.g., higher ACS in the poor-functioning group) was calculated.

#### 2.4.4. Sequential Symptom-Removal Sensitivity Analyses

Although the complete subset enumeration analyses provide a comprehensive assessment of ACS performance across all possible symptom-term combinations, they include many combinations that would be unlikely to be selected in practice. In most research settings, symptom terms are intentionally selected to reflect the anticipated toxicity profile of the target population, with preference given to symptom terms considered most relevant for that disease setting.

To evaluate ACS performance under a more realistic symptom-selection strategy, additional sensitivity analyses were conducted by sequentially removing symptom terms according to their relative relevance in previously published disease-specific symptom-selection studies [20,22,24]. Symptom terms were ordered from least to most highly prioritized based on these published rankings, and the lowest-ranked symptom term was removed at each step until only three symptom terms remained.

After each sequential removal step, the ACS was recalculated and evaluated with respect to test–retest reliability among GRC-defined stable participants, responsiveness (SRMs within GRC-defined improvement, no-change, and worsening groups), and known-groups validity using both patient-reported physical functioning (good vs. poor) and ECOG performance status (0–1 vs. 2–4). This analysis was intended to assess the extent to which ACS psychometric performance is preserved as progressively lower-priority symptom terms are excluded.

#### 2.4.5. Sensitivity to Group Differences in Longitudinal Trajectories

Sensitivity to group differences was further evaluated using the COMET-2 trial by comparing longitudinal symptomatic AE burden between treatment arms using area-under-the-curve (AUC) analyses. ACS were analyzed using repeated-measures mixed-effects models with fixed effects for treatment arm, assessment timepoint, and their interaction, and an unstructured within-participant covariance structure to account for correlation among unequally spaced repeated measurements. Model-estimated mean ACS at each timepoint for each treatment arm were obtained and integrated over time using the trapezoidal rule to estimate AUCs. Differences in AUCs (cabozantinib minus mitoxantrone–prednisone) were used to assess whether the ACS captured differential symptom trajectories between treatment groups. Sensitivity analyses were performed across varying numbers and compositions of symptomatic AE subsets using exact enumeration. All analyses were conducted using R 4.4.1, with mixed-effects models fit using the nlme package v3.1 [31]. These analyses were designed as a proof-of-concept evaluation to assess whether the ACS is capable of detecting between-group differences in overall symptom burden in a randomized trial setting, rather than as a confirmatory comparison.

#### 2.4.6. Multiplicity Adjustment

No adjustment for multiple comparisons was applied. The analyses were conducted to holistically evaluate distinct psychometric properties of the ACS, including reliability, responsiveness, known-groups validity, and sensitivity to symptom selection, rather than to formally test a single family of confirmatory hypotheses. Accordingly, *p*-values were interpreted as exploratory evidence rather than confirmatory tests. Greater emphasis was placed on the magnitude, direction, and consistency of the observed effects across main and sensitivity analyses.

## 3. Results

Table 1 shows the descriptive statistics of the PRO-CTCAE ACS or its change scores used in three psychometric analyses (i.e., test–retest reliability, responsiveness, known-groups validity analyses).

### 3.1. Test–Retest Reliability

Using the Visit 1b sample, test–retest reliability of the PRO-CTCAE ACS was high in both lung and head and neck cancer samples. In lung cancer, the ACS computed from five symptoms (shortness of breath, pain, fatigue, decreased appetite, and nausea) demonstrated excellent reliability, with an ICC of 0.88 (95% CI: 0.70–0.96). In head and neck cancer, the ACS computed from 11 symptoms (dry mouth, anxiety, constipation, decreased appetite, pain, fatigue, insomnia, mouth sores, nausea, sad/unhappy feelings, and vomiting) yielded an ICC of 0.90 (95% CI: 0.84–0.94). In both disease groups, ICC estimates exceeded the prespecified threshold for acceptable test–retest reliability (ICC ≥ 0.70).

**Figure 2:**
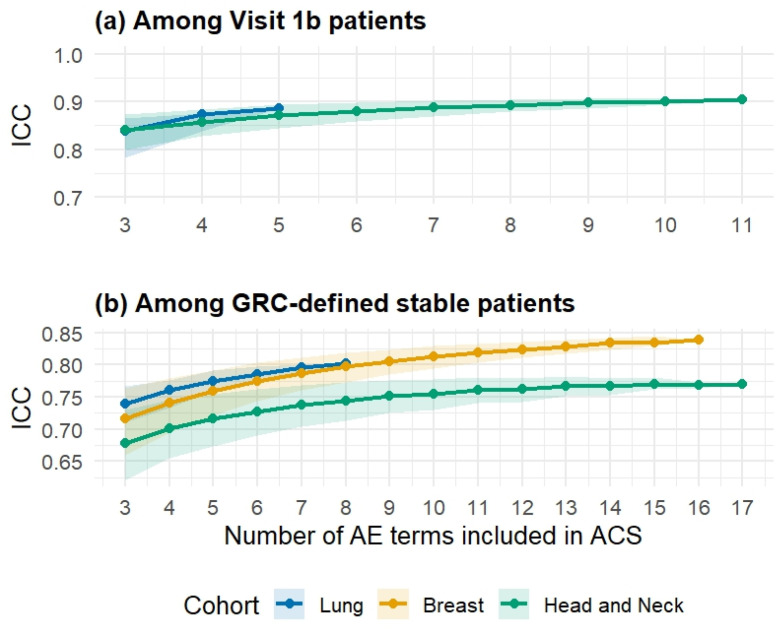
Test–Retest Reliability (ICC) of the PRO-CTCAE Average Composite Score Across Symptom Subset Sizes. *Note.* Test–retest reliability was assessed using ICCs computed from (1) assessments conducted one day apart, for which symptom status over the preceding 7 days was expected to be stable, and (2) assessments completed one to six weeks apart among participants who reported that their physical condition had remained about the same since the initial assessment. For each symptom subset size, the ACS was calculated as the mean of AE-level PRO-CTCAE composite scores. Solid lines represent the median ICC across evaluated symptom-term subsets, and shaded ribbons indicate the IQR of ICC values across subsets, reflecting the sensitivity of test–retest reliability estimates to symptom term selection.

Sensitivity analyses examining the effect of varying the number of symptom terms included in the ACS are shown in Figure 2a. In lung cancer, exact combinatorial evaluation of all possible subsets of three to five symptoms produced median ICC values of 0.84 and 0.87 when three and four symptoms were included, respectively (Table S2). In head and neck cancer, median ICC values increased from 0.84 to 0.90 as the number of included symptom terms increased from three to ten (Table S3). Across sensitivity analyses, all subsets in lung cancer and nearly all subsets in head and neck cancer met the predefined reliability criterion (ICC ≥ 0.70).

Using the GRC-defined stable patients, test–retest reliability of the PRO-CTCAE ACS exceeded the prespecified threshold (0.70). In lung cancer, the ICC of the ACS computed from eight symptoms was 0.80 (95% CI: 0.70–0.87). In breast cancer, the ICC of the ACS computed from 16 symptoms was 0.84 (95% CI: 0.77–0.89). In head and neck cancer, the ICC of the ACS computed from 17 symptoms was 0.77 (95% CI: 0.63–0.86).

Sensitivity analyses are shown in Figure 2b. In lung cancer, combinatorial evaluation of subsets ranging from three to seven symptoms yielded median ICC values between 0.74 and 0.80 (Table S4). In breast cancer, median ICC values increased from 0.72 to 0.84 as the number of included symptom terms increased from three to 15 (Table S5). In head and neck cancer, median ICC values increased from 0.68 to 0.77 as the number of symptom terms increased from three to 16 (Table S6). Reliability improved progressively with larger symptom sets, with most subsets meeting or exceeding the ICC ≥ 0.70 threshold.

### 3.2. Responsiveness

The absolute magnitude of the correlation between ACS change score and the GRC ordering was 0.24 for lung cancer, 0.29 for breast cancer, and 0.32 for head and neck cancer. Among lung cancer patients (n = 155), SRMs were 0.30, 0.15, and −0.37 for the improved (n = 50), no-change (n = 65), and worsened (n = 40) groups, respectively, with a significant monotonic trend across GRC categories (*p* = 0.002). Similar ordered patterns were observed in breast cancer (n = 208), with SRMs of 0.29, 0.14, and −0.40 for the improved (n = 55), no-change (n = 99), and worsened (n = 54) groups (*p* < 0.001). In head and neck cancer (n = 124), SRMs were −0.01, −0.20, and −0.56 for the improved (n = 27), no-change (n = 52), and worsened (n = 45) groups, respectively, also indicating a significant monotonic trend (*p* = 0.002).

**Figure 3:**
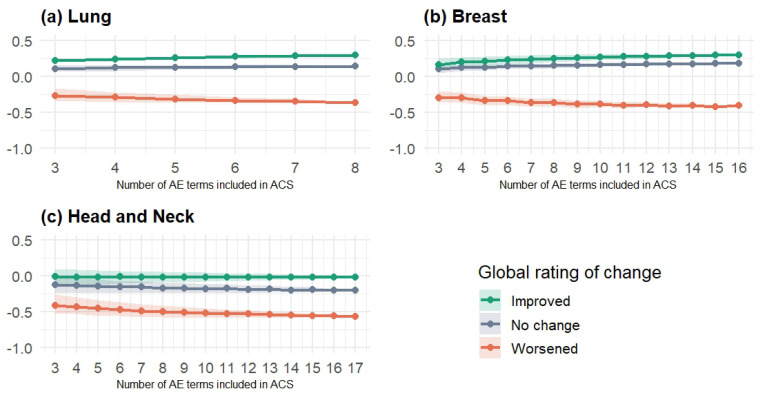
Responsiveness of the PRO-CTCAE Average Composite Score Across Symptom Subset Sizes: Standardized Response Mean (SRM). *Note.* Change scores (Visit 1 minus Visit 2) were defined such that positive values indicate symptom improvement and negative values indicate symptom worsening. Lines represent the median SRM estimated across all enumerated symptom subsets for each global rating of change category. Shaded ribbons indicate the IQR of SRM values across subsets, reflecting variability in responsiveness attributable to different selections and numbers of PRO-CTCAE symptom terms included in the ACS.

Sensitivity analyses evaluating the effect of symptom term selection on responsiveness are shown in Figure 3 and Tables S7–S9. For each cancer type, lines show the median SRM across all enumerated symptom subsets for each GRC category. Across all disease groups, median SRMs were well separated between the self-reported improved/no-change and worsened groups, with greater median separation observed as the number of symptom terms included in the ACS increased.

**Figure 4:**
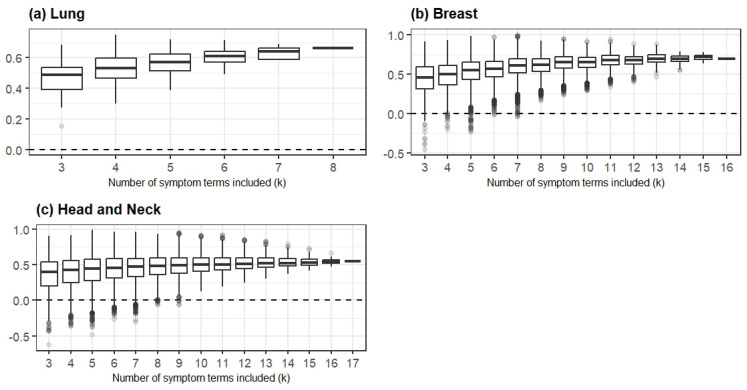
Distribution of SRM Separation (Improved–Worsened). *Note.* Boxplots show the distribution of SRM separation between patients reporting improvement and worsening across all samples for each subset size (*k*). The y-axis represents the difference in SRMs between patients reporting improvement and worsening. Larger positive values indicate greater discrimination between self-reported improved and worsened groups.

In lung cancer, when three symptom terms were included (*k* = 3), the median distance between SRMs for the improved and worsened group was 0.49, with separation increasing gradually to 0.64 when seven terms were included (Figure 4 and Table S7). Although the magnitude of separation varied depending on the specific symptom terms selected, the SRM for the improved group exceeded that of the worsened group in all subsets.

**Figure 5:**
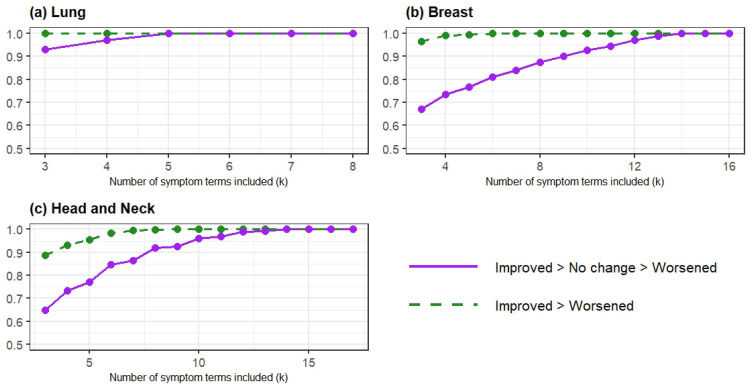
Proportion of Subsets Satisfying Expected SRM Orderings (Improved > No change > Worsened; Improved > Worsened) Across Symptom Subset Sizes. **Note.** Lines show the proportion of all enumerated subsets in which SRMs satisfied expected ordering criteria across symptom subset sizes (*k*). Purple solid lines indicate the full ordinal ordering based on the patient-reported global rating of change (Improved > No change > Worsened), while green dashed lines indicate directional separation only (Improved > Worsened).

In breast cancer, when *k* = 3, the median SRM distance between the improved and worsened groups was 0.46, increasing steadily to 0.72 when 15 symptom terms were incorporated into the ACS (Figure 4). At *k* = 3, the SRM for the improved group exceeded that of the worsened group in 96.4% of all enumerated subsets, increasing to 100% by *k* = 6 (Figure 5 and Table S8).

In head and neck cancer, when *k* = 3, the median distance between the improved and worsened groups was 0.40, increasing gradually to 0.54 when 16 symptom terms were included in the ACS (Figure 4 and Table S9). At *k* = 3, the SRM for the improved group exceeded that of the worsened group in 88.8% of enumerated subsets, increasing to 93.2% at *k* = 4, 95.4% at *k* = 5, and reaching 100% by *k* = 8 (Figure 5). Compared to lung and breast cancer, greater variability in SRM separation was observed across symptom selections at smaller subset sizes, with more frequent instances in which the SRM for the self-reported improved group was smaller than that for the worsened group, indicating reversed responsiveness. The largest such reversal (SRMi_mproved_ − SRM_worsened_ = −0.63) occurred when constipation, cough, and radiation skin reaction were the only three symptom terms included (Table S10). Conversely, the greatest separation in the expected direction at *k* = 3 (SRMi_mproved_ − SRM_worsened_ = 0.90) occurred when fatigue, dry mouth, and hoarseness were included.

Collectively, these findings demonstrate that responsiveness of the ACS is sensitive to symptom term selection when small subsets are used but becomes increasingly stable and directionally consistent as additional clinically relevant symptom terms are included. When only a few symptoms are selected, inadequate representation of patients’ most salient symptom changes may attenuate or, in rare cases, reverse observed responsiveness despite patient-reported improvement.

### 3.3. Known-Groups Validity

The EORTC dichotomized physical functioning indicator demonstrated moderate associations with the ACS (|*r*| *=* 0.41 in lung cancer, |*r*| *=* 0.61 in breast cancer, |*r*| *=* 0.49 in head and neck cancer), supporting its use as a known-groups validity anchor. The ACS demonstrated expected discrimination between EORTC-based, patient-reported physical function groups across cohorts. Mean ACS values were significantly lower among patients with good physical functioning than among those with poor physical functioning in lung cancer (0.71 vs. 1.23; *n* = 68 vs. *n* = 104; *p* < 0.001, Cohen’s *d* = 0.91 with 95% CI: 0.59–1.23), breast cancer (0.52 vs. 1.19; *n* = 137 vs. *n* = 84; *p* < 0.001, Cohen’s *d =* 1.59 with 95% CI: 1.28–1.89), and head and neck cancer (0.69 vs. 1.29; *n* = 82 vs. *n* = 56; *p* < 0.001, Cohen’s *d* = 1.14 with 95% CI: 0.77–1.50).

**Figure 6:**
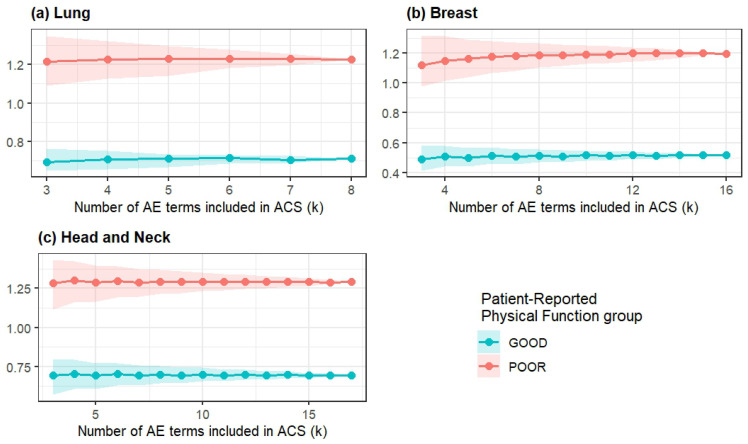
Known-Groups Validity of the PRO-CTCAE Average Composite Score Across Symptom Subset Sizes: Mean ACS by Patient-Reported Physical Functioning Status. *Note.* Known-groups validity of the PRO-CTCAE ACS was evaluated by physical functioning status (good vs. poor). For each disease group, all possible subsets of symptom terms were enumerated for each subset size (*k*) (3–8 for lung, 3–16 for breast, and 3–17 for head and neck cancers). Lines represent the median of mean ACS across all enumerated subsets for each physical functioning group, and shaded ribbons indicate the IQR of mean ACS values across subsets.

Sensitivity analyses evaluating the robustness of known-groups validity to symptom selection are shown in Figure 6. Across cancer types, median group-specific mean ACS values remained stable across subset sizes (*k*), whereas variability in group-specific means decreased as additional symptom terms were included.

Discrimination between good and poor physical function groups was completely robust across all disease cohorts, with higher mean ACS values consistently observed in the poor physical functioning group across all enumerated subsets across all subset sizes, including those containing only three symptom terms (*k* = 3). In lung cancer, the difference in mean ACS between physical functioning groups ranged from 0.22 to 0.83 across subsets, with the largest separation observed when shortness of breath, fatigue, and pain were included and the smallest when sadness, nausea, and cough were included (Table S11). In breast cancer, differences ranged from 0.26 to 1.04, with the greatest separation when fatigue, pain, and aching joints were included, and the smallest when dizziness, diarrhea, and heart palpitations were included (Table S12). In head and neck cancer, differences ranged from 0.28 to 0.85, with the largest separation observed for decreased appetite, cough, and fatigue and the smallest for insomnia, radiation-related skin reaction, and hoarseness (Table S13).

The ECOG PS dichotomized indicator was weakly correlated with the ACS (|*r*| *=* 0.27 in lung cancer, |*r*| *=* 0.22 in breast cancer, |*r*| *=* 0.10 in head and neck cancer). Consistent with these modest correlations, the ACS demonstrated weaker discrimination between ECOG-defined groups than between patient-reported physical functioning groups. Nonetheless, mean ACS values were significantly lower among patients with ECOG 0–1 than ECOG 2–4 in lung cancer (0.93 vs. 1.31; *n* = 133 vs. *n* = 41; *p* = 0.002, Cohen’s *d =* 0.65 with 95% CI: 0.29–1.00) and breast cancer (0.74 vs. 1.22; *n* = 207 vs. *n* = 15; *p* = 0.001, Cohen’s *d* = 0.91 with 95% CI: 0.38–1.44). In head and neck cancer, the direction of the difference was consistent with expectations but did not reach statistical significance (0.90 vs. 1.06; *n* = 111 vs. *n* = 28; *p* = 0.30, Cohen’s *d* = 0.26 with 95% CI: −0.16–0.67).

**Figure 7:**
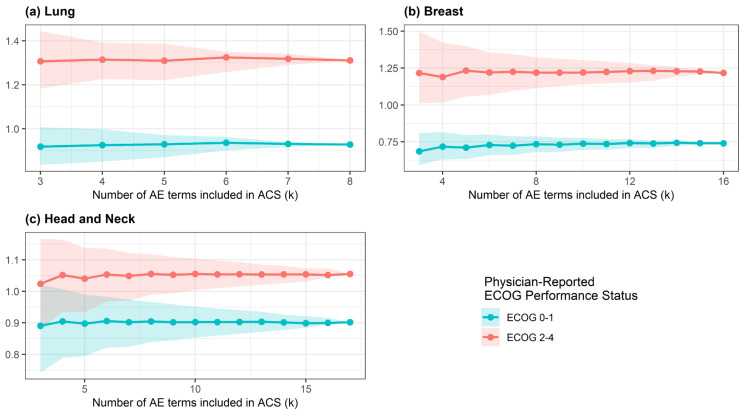
Known-Groups Validity of the PRO-CTCAE Average Composite Score Across Symptom Subset Sizes: Mean ACS by Physician-Reported ECOG Status. *Note.* Known-groups validity of the PRO-CTCAE ACS was evaluated by ECOG status (ECOG 0–1 vs. ECOG 2–4). For each disease group, all possible subsets of symptom terms were enumerated for each subset size (*k*) (3–8 for lung, 3–16 for breast, and 3–17 for head and neck cancers). Lines represent the median of mean ACS across all enumerated subsets for each ECOG PS group, and shaded ribbons indicate the IQR of mean ACS values across subsets.

Sensitivity analyses evaluating the robustness of ECOG-based known-groups validity to symptom selection are shown in Figure 7. Across cancer types, median ECOG-specific mean ACS values remained stable across subset sizes, while variability decreased as additional symptom terms were included. In lung cancer, discrimination between ECOG 0–1 and ECOG 2–4 was completely robust, with higher mean ACS values observed in the ECOG 2–4 group in all enumerated subsets (Table S14). In breast cancer, ECOG group separation was highly consistent, with reversals observed in only 3 (0.5%) of 560 subsets at *k* = 3 and 1 (0.05%) out of 1820 subsets at *k* = 4; from *k* ≥ 5 onward, higher symptom burden in the ECOG 2–4 group was observed across all subsets (Table S15). In head and neck cancer, small instability was observed at smaller subset sizes, with reversed ordering in 37 (2.9%) out of 680 subsets at *k* = 3, 42 (1.8%) out of 2380 subsets at *k* = 4, 46 (0.7%) out of 6188 subsets at *k* = 5, 15 (0.1%) out of 12376 subsets at *k* = 6, 4 (0.02%) out of 19,448 subsets at *k* = 7 and 0% reversal from *k* ≥ 8 (Table S16). Overall, these findings indicate that the PRO-CTCAE ACS demonstrates strong known-groups validity across cancer types, with increasingly stable and consistent discrimination as the number of included symptom terms grows. At small subset sizes, symptom selection greatly influences the magnitude of group differences. Although larger symptom sets yield more stable estimates, small symptom subsets are capable of achieving comparable or greater discrimination when highly informative symptom terms are selected, whereas larger subsets provided more consistent results across a wide range of clinically relevant symptom combinations.

### 3.4. Sequential Symptom-Removal Sensitivity Analyses

Tables S17–S19 summarize the sequential symptom-removal sensitivity analyses. Across all three disease cohorts, ACS psychometric performance remained largely stable as progressively lower-priority symptom terms were removed, even when the ACS was reduced to only three to five symptom terms. Test–retest reliability remained acceptable throughout symptom reduction. ICC estimates remained ≥ 0.78 across all subset sizes in the lung cohort, ≥ 0.76 in the breast cohort when *k* ≥ 4 (0.70 when *k* = 3), and ≥ 0.73 in the head and neck cohort when *k* ≥ 5 (0.69 when *k* = 4 and 0.73 when *k* = 3). Responsiveness was preserved across all symptom subsets, with statistically significant monotonic trends in ACS change across GRC-defined improvement categories (all trend test *p* < 0.05). Evidence for known-groups validity based on patient-reported physical functioning remained strong throughout symptom reduction, with Cohen’s *d* values of at least 0.91 in the lung cohort, 1.39 in the breast cohort, and 0.90 in the head and neck cohort. Known-groups validity using ECOG PS also remained strong in the lung (minimum Cohen’s *d* = 0.59) and breast (minimum Cohen’s *d* = 0.78) cohorts. Effect sizes were smaller in the head and neck cohort (Cohen’s *d* = 0.14–0.26), consistent with the weaker correlation observed between ACS and ECOG performance status in that cohort (*r* = 0.05–0.10 across subset sizes).

### 3.5. Sensitivity to Group Differences

**Figure 8:**
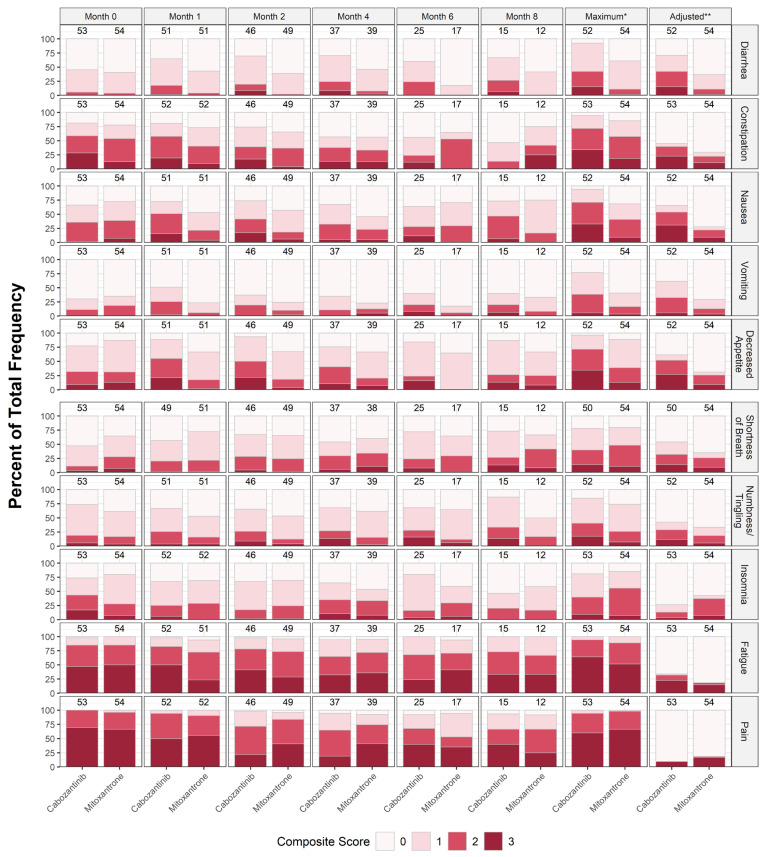
Distribution of the PRO-CTCAE Composite Scores across 10 symptomatic AE terms by treatment arm from Month 0 to Month 8. *Note.* Higher composite scores indicate worse symptoms. Column labels (n) show the number of patients with an observed symptom composite score. * Maximum composite score reported post-baseline per patient. ** Maximum score reported post-baseline per patient when including only scores that were worse than the patient’s baseline score.

**Figure 9:**
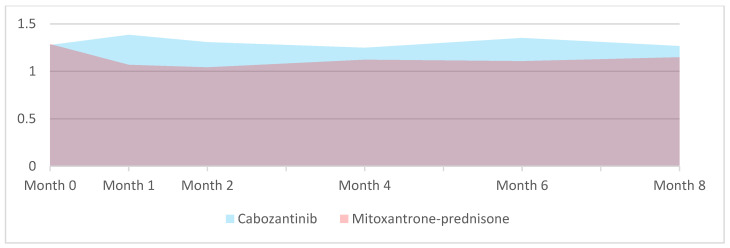
Sensitivity to Group Differences: Model-Based AUC by Treatment Arm Using a Ten-Symptom PRO-CTCAE ACS. *Note.* Curves represent model-estimated ACS trajectories over time for each treatment arm, and shaded areas indicate the corresponding area under the curves, reflecting overall symptom burden during follow-up. Higher AUC values indicate greater cumulative symptom burden. Higher overall symptom burden was observed in the cabozantinib arm (AUC = 10.2) than in the mitoxantrone–prednisone arm (AUC = 8.7), yielding a mean difference of 1.5 (95% CI: 0.2–2.8, *p* = 0.02).

**Figure 10:**
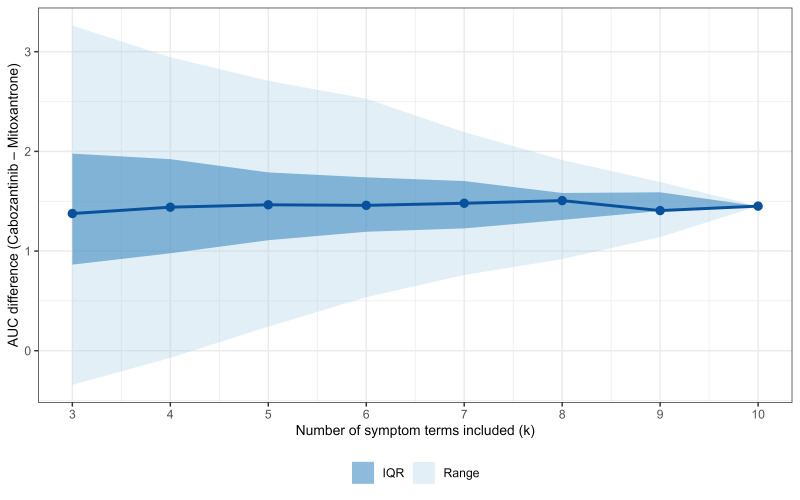
Sensitivity of Treatment Group Differences to Symptom Term Selection. *Note.* Points indicate the median difference in model-based AUC for the ACS between treatment arms across all enumerated symptom subsets of size *k*. The dark shaded ribbon represents the IQR, and the light shaded ribbons represent the full range (minimum to maximum) of AUC differences observed across subsets. Positive values indicate greater cumulative symptom burden in the cabozantinib arm relative to mitoxantrone–prednisone.

Figure 8 shows the distribution of the PRO-CTCAE composite scores for ten symptomatic AE terms from baseline to Month 8. Mixed-effects AUC analyses suggested higher overall symptom burden in the cabozantinib arm (AUC = 10.2) than in the mitoxantrone–prednisone arm (AUC = 8.7), yielding a mean difference of 1.5 (95% CI: 0.2–2.8, *p* = 0.02; Figure 9). This finding is consistent with a pattern of symptom burden observed across the individual PRO-CTCAE terms and provides supportive evidence that the ACS can summarize cumulative symptomatic AE burden in a randomized trial setting. However, the result should be interpreted cautiously given the limited sample size, early trial termination, and incomplete follow-up. Sensitivity analyses assessing robustness to symptom term selection are shown in Figure 10, which summarizes the median, IQR and range of AUC differences across all enumerated symptom subsets for each subset size (*k*). Median AUC differences were stable across values of *k*, while variability decreased as additional symptom terms were included.

When *k* = 3, the minimum observed AUC difference was −0.34 for the subset including pain, insomnia, and shortness of breath, whereas the maximum difference was 3.26 for the subset including diarrhea, decreased appetite, and nausea (Table S20). At *k* = 4, the minimum difference was −0.07 for fatigue, pain, insomnia, and shortness of breath, whereas the maximum difference was 2.94 for numbness/tingling, diarrhea, decreased appetite, and nausea. Overall, the magnitude and direction of treatment-group differences depended on the symptom composition of the ACS. Symptom subsets including shortness of breath or insomnia tended to show relatively small differences and occasionally favored the cabozantinib arm, whereas subsets containing gastrointestinal symptoms yielded larger between-group differences favoring the mitoxantrone–prednisone arm.

## 4. Discussion

### 4.1. Interpreting the ACS Within the Broader PRO-CTCAE Literature

Since its introduction, PRO-CTCAE has advanced evaluation of symptomatic adverse events in oncology by enabling direct patient reporting of symptom frequency, severity, and interference [32]. Methods for summarizing information across multiple symptoms have most often relied on threshold-based indicators or symptom-specific estimands, such as the proportion of patients reporting at least one PRO-CTCAE item or composite score exceeding a predefined threshold relative to baseline, or time-to-event analyses using symptom-specific cutpoints (e.g., time to first PRO-CTCAE pain severity score ≥3) [3]. While these approaches align with PRO-CTCAE’s role as a companion to clinician-reported CTCAE and facilitate categorical interpretation of ordinal data, the potential of PRO-CTCAE data to support scale-level representations of cumulative symptom burden has been underexplored. More recently, the Toxicity Index [33] has been adapted for PRO-CTCAE data as a summary measure [13] that prioritizes the highest-severity AE while incorporating information from lower-severity AEs to rank patients according to their maximum grade and cumulative toxicity burden. Prior methodological work has shown that the Toxicity Index-based approaches may offer greater statistical efficiency than conventional maximum-grade toxicities under certain modeling assumptions, potentially increasing sensitivity for detecting treatment-group differences [34].

While the Toxicity Index-based approach is a ranking measure, the PRO-CTCAE ACS is an estimate of average symptom burden, analogous to the mean or sum scores commonly used throughout patient-reported outcome and psychometric research. As an equally weighted summary measure shown to retain a monotonic relationship with the underlying latent symptom burden [5], the ACS provides an estimate of overall symptom burden across a prespecified set of symptomatic AEs. Prior work demonstrated strong structural validity of the ACS across multiple cancer populations [5]. However, structural validity alone is insufficient for supporting longitudinal or comparative use. The present study extends the psychometric evaluation of the ACS, in line with COSMIN and regulatory guidance, by examining additional measurement properties critical for fit-for-purpose use in oncology research.

### 4.2. Implications for Reliability, Responsiveness, and Known-Groups Validity

The ACS demonstrated strong test–retest reliability, with ICC values exceeding the acceptable thresholds. Sensitivity analyses further showed that reliability was consistently high across symptom subsets and increased as additional symptom terms were incorporated. Responsiveness analyses indicated that the ACS detects patient-defined change over time, with ordered separation across patient-reported GRC and statistically significant monotonic trends across cancer types. Sensitivity analyses revealed greater variability at small symptom subset sizes, with occasional reversals, but increasingly robust and consistent responsiveness as more symptom terms were included. This suggests small symptom subsets are more vulnerable to idiosyncratic symptom trajectories if terms are not carefully selected, whereas higher symptom coverage yields more stable assessment of overall change.

Known-groups validity analyses further demonstrated that the ACS discriminated between clinically distinct patient-reported physical functioning or physician-reported performance status groups across cancer cohorts, with discrimination becoming more stable as additional symptom terms were included. In the head and neck cohort, the weak association between ACS and ECOG performance status resulted in group differences that were directionally consistent with expectations but were characterized by a small effect size (*d =* 0.26) and did not reach statistical significance. In all other comparisons, group differences demonstrated moderate-to-large effect sizes, with Cohen’s *d* estimates ranging from 0.67 to 1.59.

Importantly, a complementary sequential symptom-removal analysis demonstrated that ACS performance remained largely preserved when symptom terms were removed according to published disease-specific relevance rankings. Test–retest reliability generally remained within acceptable ranges, SRM values for the improved, no-change, and worsened groups remained stable across all reduced symptom subsets, and known-groups validity using a patient-reported physical functioning anchor was maintained even when the ACS was calculated using a small number of symptom terms. These findings suggest that although inclusion of a broader range of symptoms improves stability on average, carefully selected high-priority symptom terms guided by established evidence regarding symptom relevance within the target population may still provide a psychometrically robust summary measure when questionnaire length or respondent burden necessitates a more parsimonious assessment strategy.

Together, these findings provide evidence supporting the reliability, responsiveness, and known-groups validity of the ACS as a summary score for both cross-sectional and longitudinal comparisons of symptomatic AE burden, when complemented by reporting of the specific profile of individual symptom-level AEs.

### 4.3. Sensitivity to Group Differences and Implications for Symptom Selection

Analyses of the COMET-2 trial suggested that the ACS was capable of capturing between-arm differences in cumulative symptom burden when evaluated using model-based AUC methods. Sensitivity analyses showed that estimated treatment differences were mostly directionally consistent across symptom subsets, while their magnitude depended on both the number and composition of symptoms included. When only a small number of symptoms are included, the sensitivity of the ACS to detect group differences depends heavily on specific selected symptoms, with symptom subsets including gastrointestinal symptoms (e.g., diarrhea, nausea, decreased appetite) producing larger AUC differences between treatment arms. Variability in ΔAUC decreased as *k* approached the full set of available symptoms, reflecting both the diminishing influence of individual symptomatic AEs and the reduced number of possible symptom combinations.

These findings are consistent with prior methodological guidance emphasizing that multisymptom composites require careful, context-specific symptom selection [35], as the relevance and expected change in individual symptoms vary substantially by cancer type, disease stage, and treatment; failure to align symptom selection with the target population and trial objectives may attenuate sensitivity and interpretability, a challenge that is ubiquitous across composite PRO endpoints rather than specific to the ACS approach [36].

This observation has direct implications for fit-for-purpose PRO design. In COMET-2, the primary clinical objective was pain palliation rather than characterization of overall symptomatic burden, making pain-specific endpoints methodologically appropriate. Conversely, when the goal is to characterize broader treatment tolerability or cumulative side-effect burden, inclusion of additional symptom domains aligned with the target population may be warranted. Given the early termination of COMET-2 and the resulting limited sample size and follow-up, the modest between-group differences observed for the ACS trajectories should be interpreted cautiously. Nevertheless, the findings provide supportive evidence that the ACS may be able to detect differences in cumulative symptom burden when such differences exist.

Several limitations inherent to summary measures should be acknowledged. As demonstrated in our prior structural validity analyses [5], a parsimonious summary measure necessarily simplifies multidimensional symptom experiences, and patients with similar ACS values may exhibit different symptom profiles. Accordingly, examination of individual symptom terms remains important when characterizing toxicity profiles and informing clinical management. Thus, the ACS should be viewed not as a replacement for symptom-specific PRO analyses, but as a complementary measure whose utility depends on alignment between symptom selection and the underlying research question. The sensitivity analyses presented here provide an empirical framework for designing ACS-based endpoints that are fit for their intended purpose.

### 4.4. Strengths, Limitations, and Methodological Considerations for Future Research

Strengths of this study include the use of large, well-characterized datasets across multiple cancer types; integration of observational validation data and randomized trial data; and comprehensive sensitivity analyses using both exact subset evaluations. Systematic examination of the effect of symptom subset size on psychometric properties and the identification of symptom composition that contributes to maximum versus minimum reliability, responsiveness, known-groups or treatment arm comparisons provide novel insight into the stability and interpretability of PRO-CTCAE summary scores.

Several limitations merit consideration. The symptom term sets were restricted to those available and previously identified as clinically relevant, and findings may differ in other disease contexts or treatment modalities. Additionally, while the ACS applies equal weighting of symptom contributions, alternative weighting schemes like latent-variable approaches warrant future investigation. Lastly, future research may extend ACS-based analyses to additional cancer populations and examine links with downstream outcomes such as treatment adherence, tolerability, patient-reported benefit, or survival.

## 5. Conclusions

The PRO-CTCAE Average Composite Score provides a reliable, responsive, and interpretable summary measure of overall symptomatic adverse event burden across multiple cancer populations. Beyond structural validity, the ACS demonstrated strong test–retest reliability, responsiveness to patient-reported change, known-groups validity, and sensitivity to treatment group differences when evaluated in appropriate contexts. Sensitivity analyses showed that psychometric stability and robustness improve as additional clinically relevant symptom terms are included, while also highlighting the importance of symptom selection aligned with specific research objectives. These findings support the ACS as a summary metric that complements item-level PRO-CTCAE analyses and facilitates interpretation of overall symptom burden in oncology clinical trials.

## Figures and Tables

**Table 1:** Descriptive statistics of the PRO-CTCAE Average Composite Score (ACS) or its change scores by psychometric analysis and disease cohort.

	Lung Cancer	Breast Cancer	Head and Neck Cancer
**Known-Groups Validity Analysis** (Visit 1 baseline ACS)	*N* = 174	*N* = 222	*N* = 139
Mean (SD)	1.02 (0.61)	0.77 (0.54)	0.93 (0.60)
Median (IQR)	0.88 (0.63, 1.50)	0.67 (0.38, 1.08)	0.82 (0.47, 1.29)
Range	0.00, 2.63	0.00, 2.33	0.00, 2.83
**Responsiveness Analysis** (Visit 1 − Visit 2: Change in ACS)	*N* = 155	*N* = 208	*N* = 124
Mean (SD)	0.02 (0.41)	0.00 (0.33)	−0.12 (0.39)
Median (IQR)	0.00 (−0.25, 0.25)	0.00 (−0.17, 0.19)	−0.08 (−0.34, 0.08)
Range	−1.25, 1.29	−1.44, 1.12	−1.33, 0.92
GRC-defined Improved Group	*n* = 50	*n* = 55	*n* = 27
Mean (SD)	0.12 (0.40)	0.10 (0.35)	−0.00 (0.33)
Median (IQR)	0.00 (−0.13, 0.34)	0.06 (−0.08, 0.25)	0.00 (−0.24, 0.13)
Range	−0.75, 1.13	−0.75, 1.12	−0.59, 0.83
GRC-defined No-Change Group	*n* = 65	*n* = 99	*n* = 52
Mean (SD)	0.06 (0.39)	0.04 (0.26)	−0.07 (0.35)
Median (IQR)	0.00 (−0.13, 0.25)	0.00 (−0.10, 0.19)	0.00 (−0.24, 0.13)
Range	−1.25, 1.29	−0.88, 0.92	−1.00, 0.82
GRC-defined Worsened Group	*n* = 40	*n* = 54	*n* = 45
Mean (SD)	−0.15 (0.42)	−0.15 (0.38)	−0.25 (0.44)
Median (IQR)	−0.13 (−0.38, 0.13)	−0.08 (−0.43, 0.08)	−0.25 (−0.47, −0.06)
Range	−1.00, 0.75	−1.44, 0.77	−1.33, 0.92
**Test–Retest Reliability Analysis** (GRC-defined No-change Group)	*N* = 65	*N* = 99	*N* = 52
Visit 1 ACS			
Mean (SD)	0.92 (0.64)	0.67 (0.50)	0.80 (0.54)
Median (IQR)	0.75 (0.38, 1.38)	0.56 (0.25, 0.92)	0.76 (0.46, 0.92)
Range	0.00, 2.63	0.00, 2.33	0.00, 2.33
Visit 2 ACS			
Mean (SD)	0.86 (0.61)	0.64 (0.44)	0.87 (0.51)
Median (IQR)	0.63 (0.38, 1.25)	0.56 (0.28, 0.94)	0.79 (0.50, 1.25)
Range	0.00, 2.38	0.00, 1.83	0.00, 2.25
**Test–Retest Reliability Analysis** (Visit 1b-defined No-change Group)	*N* = 15		*N* = 57
Visit 1 ACS			
Mean (SD)	0.76 (0.50)		0.85 (0.55)
Median (IQR)	0.80 (0.40, 1.00)		0.82 (0.45, 1.18)
Range	0.00, 1.80		0.00, 2.45
Visit 1b ACS			
Mean (SD)	0.83 (0.53)		0.78 (0.57)
Median (IQR)	0.80 (0.40, 1.10)		0.64 (0.36, 1.09)
Range	0.20, 2.00		0.00, 2.45

Note. ACS = Average Composite Score; GRC = Global Rating of Change; IQR = interquartile range. Positive change scores indicate improvement (Visit 1 − Visit 2).

## Data Availability

Restrictions apply to the availability of these data. Data from the PRO-CTCAE validation were obtained from the U.S. National Cancer Institute and are available, with the permission of the National Cancer Institute, from S.A.M. (sandra.mitchell@nih.gov). Requests regarding access to COMET-2 data should be directed to Exelixis, Inc.

## Supplementary Materials

The following supporting information can be downloaded at: https://www.mdpi.com/article/10.3390/cancers18142265/s1, Table S1. PRO-CTCAE Terms Identified as Relevant for Three Cancer Types; Table S2. Distribution of Test–Retest Reliability (ICC) of the PRO-CTCAE Average Composite Score Across Symptom Subsets, Visit 1b Lung Cancer Cohort; Table S3. Distribution of Test–Retest Reliability (ICC) of the PRO-CTCAE Average Composite Score Across Symptom Subsets, Visit 1b Head and Neck Cancer Cohort; Table S4. Distribution of Test–Retest Reliability (ICC) of the PRO-CTCAE Average Composite Score Across Symptom Subsets, GRC-defined Stable Lung Cancer Cohort; Table S5. Distribution of Test–Retest Reliability (ICC) of the PRO-CTCAE Average Composite Score Across Symptom Subsets, GRC-defined Stable Breast Cancer Cohort; Table S6. Distribution of Test–Retest Reliability (ICC) of the PRO-CTCAE Average Composite Score Across Symptom Subsets, GRC-defined Stable Head and Neck Cancer Cohort; Table S7. Distribution of standardized response mean (SRM) across all symptom subsets by subset size (*k*) and global rating of change group, Lung Cancer Cohort; Table S8. Distribution of standardized response mean (SRM) across all symptom subsets by subset size (*k*) and global rating of change group, Breast Cancer Cohort; Table S9. Distribution of standardized response mean (SRM) across all symptom subsets by subset size (*k*) and global rating of change group, Head and Neck Cancer Cohort; Table S10. Symptom term subsets associated with maximum and minimum SRM separation (Improved–Worsened) by subset size (*k*); Table S11. Distribution of differences in mean PRO-CTCAE ACS between EORTC-based physical functioning groups (Poor–Good) across all enumerated symptom subsets by subset size (k), Lung Cancer Cohort; Table S12. Distribution of differences in mean PRO-CTCAE ACS between EORTC-based physical functioning groups (Poor–Good) across all enumerated symptom subsets by subset size (*k*), Breast Cancer Cohort; Table S13. Distribution of differences in mean PRO-CTCAE ACS between EORTC-based physical functioning groups (Poor–Good) across all enumerated symptom subsets by subset size (*k*), Head and Neck Cancer Cohort; Table S14. Distribution of differences in mean PRO-CTCAE ACS between ECOG PS groups (ECOG 2–4—ECOG 0–1) across all enumerated symptom subsets by subset size (*k*), Lung Cancer Cohort; Table S15. Distribution of differences in mean PRO-CTCAE ACS between ECOG PS groups (ECOG 2–4—ECOG 0–1) across all enumerated symptom subsets by subset size (*k*), Breast Cancer Cohort; Table S16. Distribution of differences in mean PRO-CTCAE ACS between ECOG PS groups (ECOG 2–4—ECOG 0–1) across all enumerated symptom subsets by subset size (*k*), Head and Neck Cancer Cohort; Table S17. Additional sensitivity analyses examining the impact of sequentially removing terms starting from those symptom terms that were least endorsed by patients until reaching a reduced set of 3 symptom terms, Lung Cancer Cohort; Table S18. Additional sensitivity analyses examining the impact of sequentially removing terms starting from those symptom terms that were least endorsed by patients until reaching a reduced set of 3 symptom terms, Breast Cancer Cohort; Table S19. Additional sensitivity analyses examining the impact of sequentially removing terms starting from those symptom terms that were least endorsed by patients until reaching a reduced set of 3 symptom terms, Head and Neck Cancer Cohort; Table S20. Distribution of between-group differences in ACS area under the curve (ΔAUC; cabozantinib minus mitoxantrone–prednisone) across all enumerated symptom subsets by subset size (*k*).

## Abbreviations

The following abbreviations are used in this manuscript:

AE	Adverse event
PRO-CTCAE	Patient-Reported Outcomes version of the Common Terminology Criteria for Adverse Events
ACS	Average Composite Score
ECOG	Eastern Cooperative Oncology Group
IQR	Interquartile range
GRC	Global rating of change
SRM	Standardized response mean
AUC	Area under the curve
PRO	Patient-reported outcome
EORTC QLQ-C30	European Organisation for Research and Treatment of Cancer Quality of Life Group Core Questionnaire 30-item instrument
COSMIN	COnsensus-based Standards for the Selection of Health Measurement Instruments
ICC	Intraclass correlation
