# Supplementary material for: Psychometric Evaluation of the PRO-CTCAE Average Composite Score: Reliability, Responsiveness, Known-Groups Validity, and Sensitivity to Group Differences
Source: Cancers (Basel). 2026 Jul 15;18(14):2265. doi: 10.3390/cancers18142265 (PMC13406605; doi:10.3390/cancers18142265)
Supplement: Supplementary file 1 [file cancers-18-02265-s001.zip › cancers-4373230-supplementary.pdf]

Supplementary

Table S1. PRO-CTCAE Terms Identified as Relevant for Three Cancer Types

**PRO-CTCAE Symptomatic AE Terms**	**Lung**	**Breast**	**Head/Neck**
Dry mouth			X*
Difficulty swallowing			X
Mouth/throat sores			X*
Cracking at the corners of the mouth			X
Hoarseness			X
Taste changes		X	X
Decreased appetite	X*		X*
Nausea	X*	X	X*
Vomiting			X*
Constipation	X	X	X*
Diarrhea		X	
Shortness of breath	X*	X	
Cough	X		X
Swelling		X	
Heart palpitations		X	
Hair loss		X	
Radiation skin reaction			X
Numbness & tingling		X	
Dizziness		X	
Concentration		X	
Memory		X	

**PRO-CTCAE Symptomatic AE Terms**	**Lung**	**Breast**	**Head/Neck**
General Pain	X*	X	X*
Joint pain		X	
Insomnia		X	X*
Fatigue	X*	X	X*
Anxious			X*
Sad	X		X*

Note. Symptom terms for the lung, breast, and head and neck cancer cohorts were identified based on the work of Veldhuijzen et al., Günther et al., and Sandler et al., respectively. Asterisks (*) denote AE terms that were administered at Visit 1b and were included in test-retest reliability analyses using assessments collected one-day apart.

Table S2. Distribution of Test–Retest Reliability (ICC) of the PRO-CTCAE Average Composite Score Across Symptom Subsets, Visit 1b Lung Cancer Cohort

K	Number of symptom subsets	ICC (min)	ICC (25th percentile)	ICC (median)	ICC (75th percentile)	ICC (max)	Proportion ICC ≥ 0.7	Symptom subset with minimum ICC	Symptom subset with maximum ICC
3	10	0.75	0.78	0.84	0.86	0.92	1.00	Shortness of breath, Pain, Fatigue	Pain, Decreased appetite, Nausea
4	5	0.82	0.84	0.87	0.87	0.91	1.00	Shortness of breath, Pain, Fatigue, Decreased appetite	Pain, Fatigue, Decreased appetite, Nausea
5	1	0.89	0.89	0.89	0.89	0.89	1.00	All 5 symptomatic AEs	All 5 symptomatic AEs

Note. K = Subset size

Table S3. Distribution of Test–Retest Reliability (ICC) of the PRO-CTCAE Average Composite Score Across Symptom Subsets, Visit 1b Head and Neck Cancer Cohort

K	Number of symptom subsets	ICC (min)	ICC (25th percentile)	ICC (median)	ICC (75th percentile)	ICC (max)	Proportion ICC ≥ 0.7	Symptom subset with minimum ICC	Symptom subset with maximum ICC
3	165	0.66	0.80	0.84	0.87	0.92	0.98	Anxious, Insomnia, Vomiting	Pain, Fatigue, Mouth/throat sore
4	330	0.70	0.83	0.86	0.88	0.93	1.00	Anxious, Constipation, Insomnia, Vomiting	Anxious, Decreased appetite, Pain, Mouth/throat sore
5	462	0.71	0.84	0.87	0.89	0.94	1.00	Anxious, Constipation, Insomnia, Sad, Vomiting	Anxious, Decreased appetite, Pain, Fatigue, Mouth/throat sore
6	462	0.77	0.86	0.88	0.90	0.94	1.00	Anxious, Constipation, Insomnia, Nausea, Sad, Vomiting	Anxious, Decreased appetite, Pain, Fatigue, Mouth/throat sore, Nausea
7	330	0.81	0.87	0.89	0.90	0.94	1.00	Anxious, Constipation, Fatigue, Insomnia, Nausea, Sad, Vomiting	Dry mouth, Anxious, Decreased appetite, Pain, Fatigue, Mouth/throat sore, Nausea
8	165	0.84	0.88	0.89	0.90	0.93	1.00	Dry mouth, Anxious, Constipation, Fatigue, Insomnia, Nausea, Sad, Vomiting	Dry mouth, Anxious, Decreased appetite, Pain, Fatigue, Mouth/throat sore, Nausea, Sad
9	55	0.86	0.89	0.90	0.91	0.93	1.00	Dry mouth, Anxious, Constipation, Decreased appetite, Fatigue, Insomnia, Nausea, Sad, Vomiting	Dry mouth, Anxious, Decreased appetite, Pain, Fatigue, Mouth/throat sore, Nausea, Sad, Vomiting
10	11	0.88	0.89	0.90	0.91	0.92	1.00	Dry mouth, Anxious, Constipation, Decreased appetite, Pain, Fatigue, Insomnia, Nausea, Sad, Vomiting	Dry mouth, Anxious, Decreased appetite, Pain, Fatigue, Insomnia, Mouth/throat sore, Nausea, Sad, Vomiting
11	1	0.90	0.90	0.90	0.90	0.90	1.00	All 11 symptomatic AEs	All 11 symptomatic AEs

Note. K = Subset size

Table S4. Distribution of Test–Retest Reliability (ICC) of the PRO-CTCAE Average Composite Score Across Symptom Subsets, GRC-defined Stable Lung Cancer Cohort

K	Number of enumerated symptom subsets	ICC (min)	ICC (25th percentile)	ICC (median)	ICC (75th percentile)	ICC (max)	Proportion ICC ≥ 0.7	Symptom subset with minimum ICC	Symptom subset with maximum ICC
3	56	0.61	0.71	0.74	0.77	0.81	0.79	Constipation, Decreased appetite, Nausea	Decreased appetite, Shortness of breath, Cough
4	70	0.67	0.73	0.76	0.78	0.81	0.93	Constipation, Decreased appetite, Nausea, Sad	Decreased appetite, Fatigue, Shortness of breath, Cough
5	56	0.71	0.76	0.77	0.79	0.82	1.00	Constipation, Decreased appetite, Nausea, Sad, Cough	Decreased appetite, Fatigue, Pain, Shortness of breath, Cough
6	28	0.75	0.77	0.78	0.80	0.81	1.00	Constipation, Decreased appetite, Nausea, Sad, Shortness of breath, Cough	Decreased appetite, Fatigue, Nausea, Pain, Shortness of breath, Cough
7	8	0.78	0.78	0.80	0.80	0.82	1.00	Constipation, Decreased appetite, Fatigue, Nausea, Sad, Shortness of breath, Cough	Decreased appetite, Fatigue, Nausea, Pain, Sad, Shortness of breath, Cough
8	1	0.80	0.80	0.80	0.80	0.80	1.00	All 8 symptomatic AEs	All 8 symptomatic AEs

Note. K = Subset size

Table S5. Distribution of Test–Retest Reliability (ICC) of the PRO-CTCAE Average Composite Score Across Symptom Subsets, GRC-defined Stable Breast Cancer Cohort

K	Number of enumerated symptom subsets	ICC (min)	ICC (25th percentile)	ICC (median)	ICC (75th percentile)	ICC (max)	Proportion ICC ≥ 0.7	Symptom subset with minimum ICC	Symptom subset with maximum ICC
3	560	0.35	0.66	0.72	0.76	0.83	0.56	Taste changes, Dizziness, Heart palpitations	Numbness/Tingling, Concentration, Shortness of breath
4	1820	0.50	0.70	0.74	0.78	0.85	0.73	Taste changes, Dizziness, Heart palpitations, Memory	Insomnia, Nausea, Shortness of breath, Aching joints
5	4368	0.51	0.72	0.76	0.79	0.86	0.85	Hair loss, Taste changes, Dizziness, Heart palpitations, Memory	Hair loss, Insomnia, Numbness/Tingling, Concentration, Aching joints
6	8008	0.60	0.74	0.78	0.80	0.87	0.94	Constipation, Hair loss, Taste changes, Dizziness, Heart palpitations, Memory	Hair loss, Insomnia, Numbness/Tingling, Concentration, Shortness of breath, Aching joints
7	11440	0.65	0.76	0.79	0.81	0.87	0.98	Swelling, Fatigue, Diarrhea, Nausea, Taste changes, Dizziness, Heart palpitations	Swelling, Hair loss, Insomnia, Numbness/Tingling, Shortness of breath, Aching joints, Memory
8	12870	0.66	0.77	0.80	0.82	0.88	1.00	Swelling, Fatigue, Hair loss, Diarrhea, Nausea, Taste changes, Dizziness, Heart palpitations	Swelling, Hair loss, Insomnia, Numbness/Tingling, Concentration, Shortness of breath, Aching joints, Memory
9	11440	0.69	0.79	0.81	0.82	0.87	1.00	Swelling, Fatigue, Hair loss, Diarrhea, Nausea, Taste changes, Dizziness, Heart palpitations, Memory	Swelling, Constipation, Hair loss, Insomnia, Numbness/Tingling, Concentration, Shortness of breath, Aching joints, Memory
10	8008	0.71	0.79	0.81	0.83	0.88	1.00	Swelling, Constipation, Fatigue, Hair loss, Diarrhea, Nausea, Taste changes, Dizziness, Heart palpitations, Memory	Swelling, Constipation, Hair loss, Insomnia, Numbness/Tingling, Pain, Concentration, Shortness of breath, Aching joints, Dizziness
11	4368	0.74	0.80	0.82	0.83	0.88	1.00	Swelling, Constipation, Fatigue, Hair loss, Diarrhea, Nausea, Numbness/Tingling, Taste changes, Dizziness, Heart palpitations, Memory	Swelling, Constipation, Hair loss, Insomnia, Numbness/Tingling, Pain, Concentration, Shortness of breath, Aching joints, Dizziness, Memory
12	1820	0.76	0.81	0.82	0.84	0.87	1.00	Swelling, Constipation, Fatigue, Hair loss, Diarrhea, Nausea, Numbness/Tingling, Taste changes, Shortness of breath, Dizziness, Heart palpitations, Memory	Swelling, Constipation, Hair loss, Insomnia, Nausea, Numbness/Tingling, Pain, Concentration, Shortness of breath, Aching joints, Dizziness, Memory
13	560	0.78	0.82	0.83	0.84	0.87	1.00	Swelling, Constipation, Fatigue, Hair loss, Diarrhea, Nausea, Numbness/Tingling,	Swelling, Constipation, Fatigue, Hair loss, Insomnia, Numbness/Tingling,

								Taste changes, Shortness of breath, Aching joints, Dizziness, Heart palpitations, Memory	Pain, Concentration, Shortness of breath, Aching joints, Dizziness, Heart palpitations, Memory
14	120	0.81	0.82	0.83	0.84	0.86	1.00	Swelling, Constipation, Fatigue, Hair loss, Insomnia, Diarrhea, Nausea, Numbness/Tingling, Concentration, Taste changes, Shortness of breath, Dizziness, Heart palpitations, Memory	Swelling, Constipation, Fatigue, Hair loss, Insomnia, Nausea, Numbness/Tingling, Pain, Concentration, Shortness of breath, Aching joints, Dizziness, Heart palpitations, Memory
15	16	0.82	0.83	0.83	0.84	0.85	1.00	Swelling, Constipation, Fatigue, Hair loss, Insomnia, Diarrhea, Nausea, Numbness/Tingling, Concentration, Taste changes, Shortness of breath, Aching joints, Dizziness, Heart palpitations, Memory	Swelling, Constipation, Fatigue, Hair loss, Insomnia, Nausea, Numbness/Tingling, Pain, Concentration, Taste changes, Shortness of breath, Aching joints, Dizziness, Heart palpitations, Memory
16	1	0.84	0.84	0.84	0.84	0.84	1.00	All 16 symptomatic AEs	All 16 symptomatic AEs

Note. K = Subset size

Table S6. Distribution of Test–Retest Reliability (ICC) of the PRO-CTCAE Average Composite Score Across Symptom Subsets, GRC-defined Stable Head and Neck Cancer Cohort

K	Number of enumerated symptom subsets	ICC (min)	ICC (25th percentile)	ICC (median)	ICC (75th percentile)	ICC (max)	Proportion ICC ≥ 0.7	Symptom subset with minimum ICC	Symptom subset with maximum ICC
3	680	0.25	0.62	0.68	0.73	0.85	0.40	Anxious, Radiation skin reaction, Cracking at corners of mouth	Fatigue, Sad, Hoarseness
4	2380	0.43	0.66	0.70	0.74	0.85	0.51	Decreased appetite, Difficulty swallowing, Radiation skin reaction, Cracking at corners of mouth	Fatigue, Sad, Hoarseness, Cracking at corners of mouth
5	6188	0.44	0.67	0.72	0.75	0.86	0.61	Anxious, Decreased appetite, Difficulty swallowing, Radiation skin reaction, Cracking at corners of mouth	Fatigue, Sad, Vomiting, Hoarseness, Cracking at corners of mouth
6	12376	0.51	0.69	0.73	0.76	0.87	0.69	Decreased appetite, Pain, Difficulty swallowing, Hoarseness, Radiation skin reaction, Cracking at corners of mouth	Fatigue, Insomnia, Sad, Cough, Hoarseness, Cracking at corners of mouth
7	19448	0.49	0.70	0.74	0.77	0.86	0.77	Anxious, Decreased appetite, Pain, Difficulty swallowing, Hoarseness, Radiation skin reaction, Cracking at corners of mouth	Fatigue, Insomnia, Sad, Vomiting, Cough, Hoarseness, Cracking at corners of mouth
8	24310	0.56	0.71	0.74	0.77	0.86	0.83	Anxious, Decreased appetite, Pain, Cough, Difficulty swallowing, Hoarseness, Radiation skin reaction, Cracking at corners of mouth	Constipation, Fatigue, Insomnia, Sad, Vomiting, Cough, Hoarseness, Cracking at corners of mouth
9	24310	0.56	0.72	0.75	0.78	0.86	0.89	Anxious, Decreased appetite, Nausea, Pain, Cough, Difficulty swallowing, Hoarseness, Radiation skin reaction, Cracking at corners of mouth	Constipation, Fatigue, Insomnia, Sad, Vomiting, Cough, Difficulty swallowing, Hoarseness, Cracking at corners of mouth
10	19448	0.61	0.73	0.76	0.78	0.84	0.92	Anxious, Decreased appetite, Insomnia, Mouth/throat sore, Pain, Vomiting, Difficulty swallowing, Hoarseness, Radiation skin reaction, Cracking at corners of mouth	Anxious, Constipation, Fatigue, Insomnia, Sad, Vomiting, Cough, Difficulty swallowing, Hoarseness, Cracking at corners of mouth
11	12376	0.64	0.74	0.76	0.78	0.83	0.97	Anxious, Constipation, Decreased appetite, Mouth/throat sore, Pain, Taste changes, Vomiting, Difficulty swallowing, Hoarseness, Radiation skin reaction, Cracking at corners of mouth	Anxious, Constipation, Dry mouth, Fatigue, Insomnia, Mouth/throat sore, Nausea, Sad, Vomiting, Cough, Cracking at corners of mouth
12	6188	0.65	0.74	0.76	0.78	0.83	0.98	Anxious, Constipation, Decreased appetite, Insomnia, Mouth/throat sore, Pain, Taste	Anxious, Constipation, Dry mouth, Fatigue, Insomnia, Nausea, Sad,

								changes, Vomiting, Difficulty swallowing, Hoarseness, Radiation skin reaction, Cracking at corners of mouth	Vomiting, Cough, Difficulty swallowing, Hoarseness, Cracking at corners of mouth
13	2380	0.67	0.75	0.77	0.78	0.82	0.99	Anxious, Constipation, Decreased appetite, Insomnia, Mouth/throat sore, Pain, Taste changes, Vomiting, Cough, Difficulty swallowing, Hoarseness, Radiation skin reaction, Cracking at corners of mouth	Anxious, Constipation, Dry mouth, Fatigue, Insomnia, Mouth/throat sore, Nausea, Sad, Vomiting, Cough, Difficulty swallowing, Hoarseness, Cracking at corners of mouth
14	680	0.69	0.75	0.77	0.78	0.81	1.00	Anxious, Constipation, Decreased appetite, Insomnia, Mouth/throat sore, Nausea, Pain, Taste changes, Vomiting, Cough, Difficulty swallowing, Hoarseness, Radiation skin reaction, Cracking at corners of mouth	Anxious, Constipation, Dry mouth, Fatigue, Insomnia, Mouth/throat sore, Nausea, Taste changes, Sad, Vomiting, Cough, Difficulty swallowing, Hoarseness, Cracking at corners of mouth
15	136	0.72	0.76	0.77	0.78	0.80	1.00	Anxious, Constipation, Decreased appetite, Dry mouth, Insomnia, Mouth/throat sore, Nausea, Pain, Taste changes, Vomiting, Cough, Difficulty swallowing, Hoarseness, Radiation skin reaction, Cracking at corners of mouth	Anxious, Constipation, Dry mouth, Fatigue, Insomnia, Mouth/throat sore, Nausea, Taste changes, Sad, Vomiting, Cough, Difficulty swallowing, Hoarseness, Radiation skin reaction, Cracking at corners of mouth
16	17	0.73	0.76	0.77	0.77	0.79	1.00	Anxious, Constipation, Decreased appetite, Dry mouth, Insomnia, Mouth/throat sore, Nausea, Pain, Taste changes, Sad, Vomiting, Cough, Difficulty swallowing, Hoarseness, Radiation skin reaction, Cracking at corners of mouth	Anxious, Constipation, Dry mouth, Fatigue, Insomnia, Mouth/throat sore, Nausea, Pain, Taste changes, Sad, Vomiting, Cough, Difficulty swallowing, Hoarseness, Radiation skin reaction, Cracking at corners of mouth
17	1	0.77	0.77	0.77	0.77	0.77	1.00	All 17 symptomatic AEs	All 17 symptomatic AEs

Note. K = Subset size

Table S7. Distribution of standardized response mean (SRM) across all symptom subsets by subset size *(k)* and global rating of change group, Lung Cancer Cohort

K	Number of enumerated symptom subsets	GRC group	SRM (min)	SRM (25th percentile)	SRM (median)	SRM (75th percentile)	SRM (max)	Median separation between Improved and Worsened	Proportion of Improved > Worsened	Proportion of Improved > No Change > Worsened
3	56	Worsened	-0.47	-0.34	-0.27	-0.17	0.11	0.49	92.9%	92.9%
		No change	-0.03	0.07	0.11	0.15	0.27			
		Improved	0.14	0.20	0.22	0.25	0.30			
4	70	Worsened	-0.51	-0.35	-0.29	-0.22	-0.06	0.53	97.1%	97.1%
		No change	0.01	0.08	0.12	0.16	0.25			
		Improved	0.17	0.22	0.24	0.27	0.31			
5	56	Worsened	-0.48	-0.36	-0.32	-0.26	-0.15	0.57	100%	100%
		No change	0.05	0.10	0.13	0.16	0.23			
		Improved	0.21	0.24	0.26	0.28	0.31			
6	28	Worsened	-0.46	-0.37	-0.33	-0.29	-0.24	0.61	100%	100%
		No change	0.08	0.11	0.14	0.16	0.21			
		Improved	0.23	0.25	0.28	0.29	0.31			
7	8	Worsened	-0.42	-0.37	-0.34	-0.31	-0.30	0.64	100%	100%
		No change	0.11	0.12	0.14	0.16	0.18			
		Improved	0.25	0.28	0.29	0.29	0.31			
8	1	Worsened	-0.37	-0.37	-0.37	-0.37	-0.37	0.66	100%	100%
		No change	0.15	0.15	0.15	0.15	0.15			
		Improved	0.30	0.30	0.30	0.30	0.30			

Note. K = Subset size

Table S8. Distribution of standardized response mean (SRM) across all symptom subsets by subset size *(k)* and global rating of change group, Breast Cancer Cohort

K	Number of enumerated symptom subsets	GRC group	SRM (min)	SRM (25th percentile)	SRM (median)	SRM (75th percentile)	SRM (max)	Median separation between Improved and Worsened	Proportion of Improved > Worsened	Proportion of Improved > No Change > Worsened (monotonic ordering)
3	560	Worsened	-0.60	-0.37	-0.29	-0.21	0.23	0.46	96.4%	67.0%
		No change	-0.11	0.05	0.10	0.14	0.24			
		Improved	-0.48	0.07	0.17	0.25	0.45			
4	1820	Worsened	-0.55	-0.37	-0.30	-0.23	0.13	0.50	99.2%	73.5%
		No change	-0.10	0.07	0.12	0.16	0.27			
		Improved	-0.37	0.12	0.20	0.27	0.46			
5	4368	Worsened	-0.60	-0.40	-0.34	-0.27	0.09	0.55	99.4%	76.7%
		No change	-0.08	0.08	0.13	0.16	0.26			
		Improved	-0.42	0.14	0.21	0.28	0.50			
6	8008	Worsened	-0.58	-0.40	-0.34	-0.28	0.03	0.57	100%	81.0%
		No change	-0.06	0.10	0.14	0.17	0.27			
		Improved	-0.27	0.17	0.23	0.29	0.49			
7	11440	Worsened	-0.62	-0.43	-0.37	-0.31	-0.03	0.61	100%	84.0%
		No change	-0.06	0.11	0.14	0.18	0.27			
		Improved	-0.28	0.18	0.24	0.30	0.50			
8	12870	Worsened	-0.61	-0.41	-0.37	-0.31	-0.07	0.62	100%	87.5%
		No change	0.00	0.12	0.15	0.18	0.28			
		Improved	-0.03	0.20	0.25	0.30	0.47			
9	11440	Worsened	-0.63	-0.44	-0.39	-0.34	-0.13	0.65	100%	90.1%
		No change	0.02	0.12	0.15	0.18	0.27			
		Improved	0.00	0.21	0.26	0.30	0.48			
10	8008	Worsened	-0.60	-0.42	-0.38	-0.34	-0.17	0.65	100%	92.7%
		No change	0.05	0.13	0.16	0.19	0.27			
		Improved	0.06	0.23	0.27	0.31	0.45			
11	4368	Worsened	-0.62	-0.45	-0.40	-0.36	-0.21	0.68	100%	94.5%
		No change	0.07	0.14	0.16	0.19	0.25			
		Improved	0.08	0.24	0.27	0.31	0.45			
12	1820	Worsened	-0.57	-0.43	-0.39	-0.36	-0.25	0.68	100%	97.1%
		No change	0.10	0.15	0.17	0.19	0.25			
		Improved	0.13	0.25	0.28	0.31	0.40			
13	560	Worsened	-0.56	-0.45	-0.41	-0.38	-0.28	0.70	100%	98.8%

		No change	0.12	0.15	0.17	0.19	0.24		
		Improved	0.18	0.26	0.28	0.31	0.38		
14	120	Worsened	-0.47	-0.42	-0.40	-0.38	-0.32	0.70	100%	100%
		No change	0.13	0.16	0.17	0.19	0.23			
		Improved	0.21	0.27	0.29	0.31	0.36			
15	16	Worsened	-0.46	-0.43	-0.42	-0.40	-0.36	0.72	100%	100%
		No change	0.16	0.17	0.18	0.18	0.22			
		Improved	0.25	0.28	0.29	0.31	0.33			
16	1	Worsened	-0.40	-0.40	-0.40	-0.40	-0.40	0.69	100%	100%
		No change	0.18	0.18	0.18	0.18	0.18			
		Improved	0.29	0.29	0.29	0.29	0.29			

Note. K = Subset size

Table S9. Distribution of standardized response mean (SRM) across all symptom subsets by subset size *(k)* and global rating of change group, Head and Neck Cancer Cohort

K	Number of enumerated symptom subsets	GRC group	SRM (min)	SRM (25th percentile)	SRM (median)	SRM (75th percentile)	SRM (max)	Median separation between Improved and Worsened	Proportion of Improved > Worsened	Proportion of Improved > No Change > Worsened (monotonic ordering)
3	680	Worsened	-0.92	-0.51	-0.41	-0.26	0.18	0.40	88.8%	64.9%
		No change	-0.68	-0.23	-0.12	0.00	0.34			
		Improved	-0.76	-0.14	-0.01	0.10	0.43			
4	2380	Worsened	-0.89	-0.53	-0.43	-0.30	0.14	0.42	93.2%	73.3%
		No change	-0.61	-0.23	-0.13	-0.04	0.31			
		Improved	-0.62	-0.11	-0.01	0.09	0.38			
5	6188	Worsened	-0.96	-0.55	-0.45	-0.33	0.08	0.44	95.4%	77.0%
		No change	-0.57	-0.24	-0.14	-0.05	0.29			
		Improved	-0.71	-0.12	-0.01	0.08	0.39			
6	12376	Worsened	-0.90	-0.56	-0.47	-0.36	0.02	0.46	98.5%	84.7%
		No change	-0.52	-0.24	-0.15	-0.07	0.29			
		Improved	-0.62	-0.10	-0.01	0.07	0.38			
7	19448	Worsened	-0.94	-0.57	-0.48	-0.39	-0.04	0.47	99.4%	86.5%
		No change	-0.51	-0.23	-0.15	-0.07	0.25			
		Improved	-0.61	-0.10	-0.01	0.06	0.35			
8	24310	Worsened	-0.89	-0.57	-0.49	-0.41	-0.10	0.48	100%	91.8%
		No change	-0.46	-0.24	-0.17	-0.09	0.21			
		Improved	-0.49	-0.09	-0.01	0.06	0.31			
9	24310	Worsened	-0.90	-0.58	-0.50	-0.43	-0.15	0.49	100%	92.6%
		No change	-0.44	-0.23	-0.17	-0.10	0.17			
		Improved	-0.46	-0.08	-0.01	0.05	0.29			
10	19448	Worsened	-0.85	-0.58	-0.51	-0.45	-0.22	0.50	100%	96.1%
		No change	-0.41	-0.24	-0.18	-0.12	0.12			
		Improved	-0.37	-0.08	-0.01	0.05	0.27			
11	12376	Worsened	-0.83	-0.58	-0.52	-0.46	-0.28	0.50	100%	96.9%
		No change	-0.39	-0.23	-0.17	-0.11	0.09			
		Improved	-0.32	-0.07	-0.01	0.04	0.23			
12	6188	Worsened	-0.79	-0.58	-0.53	-0.48	-0.33	0.51	100%	99.0%
		No change	-0.37	-0.23	-0.19	-0.13	0.05			
		Improved	-0.27	-0.06	-0.01	0.04	0.20			
13	2380	Worsened	-0.74	-0.59	-0.54	-0.49	-0.37	0.52	100%	99.3%
		No change	-0.34	-0.23	-0.18	-0.13	0.02			

		Improved	-0.23	-0.06	-0.01	0.03	0.16			
14	680	Worsened	-0.71	-0.58	-0.54	-0.51	-0.42	0.52	100%	100%
		No change	-0.31	-0.23	-0.20	-0.15	-0.04			
		Improved	-0.18	-0.05	-0.01	0.02	0.12			
15	136	Worsened	-0.67	-0.58	-0.55	-0.52	-0.47	0.53	100%	100%
		No change	-0.27	-0.22	-0.19	-0.15	-0.07			
		Improved	-0.12	-0.04	-0.01	0.02	0.08			
16	17	Worsened	-0.64	-0.57	-0.56	-0.54	-0.51	0.54	100%	100%
		No change	-0.24	-0.21	-0.20	-0.19	-0.13			
		Improved	-0.06	-0.03	-0.01	0.01	0.04			
17	1	Worsened	-0.56	-0.56	-0.56	-0.56	-0.56	0.55	100%	100%
		No change	-0.20	-0.20	-0.20	-0.20	-0.20			
		Improved	-0.01	-0.01	-0.01	-0.01	-0.01			

Note. K = Subset size

Table S10. Symptom term subsets associated with maximum and minimum SRM separation (Improved – Worsened) by subset size *(k)*

		Lung Cohort		Breast Cohort		Head and Neck Cohort	
K	Extreme (Maximum / Minimum)	Δ SRM (Improved – Worsened)	Symptom subset	Δ in SRM	Symptom subset	Δ in SRM	Symptom subset
3	Maximum separation	0.69	Constipation; Fatigue; Sad	0.92	Fatigue; Diarrhea; Nausea	0.90	Dry mouth; Fatigue; Hoarseness
	Minimum separation	0.15	Decreased appetite; Shortness of breath; Cough	-0.46	Numbness/Tingling; Heart palpitations; Memory	-0.63	Constipation; Cough; Radiation skin reaction
4	Maximum separation	0.75	Constipation; Fatigue; Pain; Sad	0.93	Fatigue; Diarrhea; Nausea; Taste changes	0.91	Dry mouth; Fatigue; Difficulty swallowing; Hoarseness
	Minimum separation	0.30	Decreased appetite; Sad; Shortness of breath; Cough	-0.20	Hair loss; Dizziness; Heart palpitations; Memory	-0.36	Constipation; Insomnia; Pain; Cough
5	Maximum separation	0.72	Constipation; Fatigue; Pain; Sad; Shortness of breath	0.98	Swelling; Fatigue; Diarrhea; Nausea; Taste changes	0.98	Dry mouth; Fatigue; Nausea; Difficulty swallowing; Hoarseness
	Minimum separation	0.39	Decreased appetite; Nausea; Sad; Shortness of breath; Cough	-0.23	Hair loss; Shortness of breath; Dizziness; Heart palpitations; Memory	-0.49	Constipation; Insomnia; Cough; Radiation skin reaction; Cracking at corners of mouth (cheilosis/cheilitis)
6	Maximum separation	0.72	Constipation; Fatigue; Nausea; Pain; Sad; Shortness of breath	0.97	Swelling; Fatigue; Diarrhea; Nausea; Taste changes; Aching joints	0.96	Dry mouth; Fatigue; Nausea; Difficulty swallowing; Hoarseness; Cracking at corners of mouth (cheilosis/cheilitis)
	Minimum separation	0.49	Decreased appetite; Fatigue; Nausea; Sad; Shortness of breath; Cough	-0.02	Hair loss; Numbness/Tingling; Aching joints; Dizziness; Heart palpitations; Memory	-0.27	Constipation; Insomnia; Pain; Taste changes; Cough; Radiation skin reaction
7	Maximum separation	0.69	Constipation; Fatigue; Nausea; Pain; Sad; Shortness of breath; Cough	1.00	Swelling; Fatigue; Diarrhea; Nausea; Numbness/Tingling; Taste changes; Aching joints	0.96	Dry mouth; Fatigue; Nausea; Taste changes; Difficulty swallowing; Hoarseness; Cracking at corners of mouth (cheilosis/cheilitis)
	Minimum separation	0.59	Constipation; Decreased appetite; Nausea; Pain; Sad; Shortness of breath; Cough	-0.05	Hair loss; Numbness/Tingling; Shortness of breath; Aching joints; Dizziness; Heart palpitations; Memory	-0.30	Constipation; Insomnia; Vomiting; Cough; Hoarseness; Radiation skin reaction; Cracking at corners of mouth (cheilosis/cheilitis)
8	Maximum separation	0.66	All 8 AE terms	0.93	Swelling; Fatigue; Diarrhea; Nausea; Pain; Taste changes; Shortness of breath; Aching joints	0.93	Anxious; Dry mouth; Fatigue; Nausea; Taste changes; Vomiting; Difficulty swallowing; Hoarseness
	Minimum separation			0.16	Swelling; Hair loss; Numbness/Tingling; Concentration; Shortness of breath; Dizziness; Heart palpitations; Memory	-0.06	Constipation; Insomnia; Vomiting; Cough; Difficulty swallowing; Hoarseness; Radiation skin reaction; Cracking at corners of mouth (cheilosis/cheilitis)
9	Maximum separation			0.95	Swelling; Fatigue; Hair loss; Diarrhea; Nausea; Numbness/Tingling; Pain; Taste changes; Aching joints	0.95	Anxious; Dry mouth; Fatigue; Nausea; Taste changes; Cough; Difficulty swallowing; Hoarseness; Cracking at corners of mouth (cheilosis/cheilitis)

	Minimum separation	0.24	Swelling; Hair loss; Numbness/Tingling; Concentration; Shortness of breath; Aching joints; Dizziness; Heart palpitations; Memory	-0.06	Constipation; Fatigue; Insomnia; Vomiting; Cough; Difficulty swallowing; Hoarseness; Radiation skin reaction; Cracking at corners of mouth (cheilosis/cheilitis)
10	Maximum separation	0.92	Swelling; Constipation; Fatigue; Insomnia; Diarrhea; Nausea; Numbness/Tingling; Pain; Concentration; Taste changes	0.91	Anxious; Dry mouth; Fatigue; Nausea; Taste changes; Cough; Difficulty swallowing; Hoarseness; Radiation skin reaction; Cracking at corners of mouth (cheilosis/cheilitis)
	Minimum separation	0.29	Swelling; Constipation; Hair loss; Numbness/Tingling; Concentration; Shortness of breath; Aching joints; Dizziness; Heart palpitations; Memory	0.13	Anxious; Constipation; Insomnia; Mouth/throat sore; Pain; Taste changes; Vomiting; Cough; Radiation skin reaction; Cracking at corners of mouth (cheilosis/cheilitis)
11	Maximum separation	0.94	Swelling; Constipation; Fatigue; Insomnia; Diarrhea; Nausea; Numbness/Tingling; Pain; Concentration; Taste changes; Aching joints	0.91	Anxious; Dry mouth; Fatigue; Nausea; Taste changes; Sad; Cough; Difficulty swallowing; Hoarseness; Radiation skin reaction; Cracking at corners of mouth (cheilosis/cheilitis)
	Minimum separation	0.34	Swelling; Constipation; Hair loss; Insomnia; Numbness/Tingling; Concentration; Shortness of breath; Aching joints; Dizziness; Heart palpitations; Memory	0.19	Anxious; Constipation; Insomnia; Mouth/throat sore; Nausea; Pain; Taste changes; Vomiting; Cough; Radiation skin reaction; Cracking at corners of mouth (cheilosis/cheilitis)
12	Maximum separation	0.88	Swelling; Constipation; Fatigue; Hair loss; Insomnia; Diarrhea; Nausea; Numbness/Tingling; Pain; Concentration; Taste changes; Aching joints	0.85	Anxious; Dry mouth; Fatigue; Nausea; Pain; Taste changes; Sad; Vomiting; Difficulty swallowing; Hoarseness; Radiation skin reaction; Cracking at corners of mouth (cheilosis/cheilitis)
	Minimum separation	0.40	Swelling; Constipation; Hair loss; Insomnia; Diarrhea; Numbness/Tingling; Concentration; Shortness of breath; Aching joints; Dizziness; Heart palpitations; Memory	0.25	Anxious; Constipation; Insomnia; Mouth/throat sore; Nausea; Pain; Taste changes; Sad; Vomiting; Cough; Radiation skin reaction; Cracking at corners of mouth (cheilosis/cheilitis)
13	Maximum separation	0.89	Swelling; Constipation; Fatigue; Hair loss; Insomnia; Diarrhea; Nausea; Numbness/Tingling; Pain; Concentration; Taste changes; Aching joints; Dizziness	0.82	Anxious; Dry mouth; Fatigue; Nausea; Pain; Taste changes; Sad; Vomiting; Cough; Difficulty swallowing; Hoarseness; Radiation skin reaction; Cracking at corners of mouth (cheilosis/cheilitis)
	Minimum separation	0.47	Swelling; Constipation; Hair loss; Insomnia; Diarrhea; Numbness/Tingling; Pain; Concentration; Shortness of breath; Aching joints; Dizziness; Heart palpitations; Memory	0.30	Anxious; Constipation; Decreased appetite; Insomnia; Mouth/throat sore; Nausea; Pain; Taste changes; Sad; Vomiting; Cough; Radiation skin reaction; Cracking at corners of mouth (cheilosis/cheilitis)
14	Maximum separation	0.79	Swelling; Constipation; Fatigue; Hair loss; Insomnia; Diarrhea; Nausea; Numbness/Tingling; Pain; Taste changes; Shortness of breath; Aching joints; Dizziness; Memory	0.79	Anxious; Decreased appetite; Dry mouth; Fatigue; Nausea; Pain; Taste changes; Sad; Vomiting; Cough; Difficulty swallowing; Hoarseness; Radiation skin reaction; Cracking at corners of mouth (cheilosis/cheilitis)

	Minimum separation			0.55	Swelling; Constipation; Fatigue; Hair loss; Insomnia; Diarrhea; Numbness/Tingling; Pain; Concentration; Shortness of breath; Aching joints; Dizziness; Heart palpitations; Memory	0.36	Anxious; Constipation; Decreased appetite; Insomnia; Mouth/throat sore; Nausea; Pain; Taste changes; Sad; Vomiting; Cough; Hoarseness; Radiation skin reaction; Cracking at corners of mouth (cheilosis/cheilitis)
15	Maximum separation			0.78	Swelling; Constipation; Fatigue; Hair loss; Insomnia; Diarrhea; Nausea; Numbness/Tingling; Pain; Concentration; Taste changes; Shortness of breath; Aching joints; Dizziness; Memory	0.72	Anxious; Decreased appetite; Dry mouth; Fatigue; Mouth/throat sore; Nausea; Pain; Taste changes; Sad; Vomiting; Cough; Difficulty swallowing; Hoarseness; Radiation skin reaction; Cracking at corners of mouth (cheilosis/cheilitis)
	Minimum separation			0.63	Swelling; Constipation; Fatigue; Hair loss; Insomnia; Diarrhea; Nausea; Numbness/Tingling; Pain; Concentration; Shortness of breath; Aching joints; Dizziness; Heart palpitations; Memory	0.42	Anxious; Constipation; Decreased appetite; Insomnia; Mouth/throat sore; Nausea; Pain; Taste changes; Sad; Vomiting; Cough; Difficulty swallowing; Hoarseness; Radiation skin reaction; Cracking at corners of mouth (cheilosis/cheilitis)
16	Maximum separation			0.69	All 16 AE terms	0.66	Anxious; Decreased appetite; Dry mouth; Fatigue; Insomnia; Mouth/throat sore; Nausea; Pain; Taste changes; Sad; Vomiting; Cough; Difficulty swallowing; Hoarseness; Radiation skin reaction; Cracking at corners of mouth (cheilosis/cheilitis)
	Minimum separation					0.47	Anxious; Constipation; Decreased appetite; Fatigue; Insomnia; Mouth/throat sore; Nausea; Pain; Taste changes; Sad; Vomiting; Cough; Difficulty swallowing; Hoarseness; Radiation skin reaction; Cracking at corners of mouth (cheilosis/cheilitis)
17	Maximum separation					0.55	All 17 AE terms

Note. K = Subset size. ΔSRM denotes the difference in standardized response mean between the improved and worsened groups (Improved − Worsened). Maximum and minimum values reflect the symptom subsets yielding the greatest and least separation, respectively. Negative values indicate reversal of the expected ordering (SRM for worsened exceeding SRM for improved).

Table S11. Distribution of differences in mean PRO-CTCAE ACS between EORTC-based physical functioning groups (Poor – Good) across all enumerated symptom subsets by subset size *(k)*, Lung Cancer Cohort

K	Number of enumerated symptom subsets	ΔACS (min)	ΔACS (25th percentile)	ΔACS (median)	ΔACS (75th percentile)	ΔACS (max)	Proportion (ΔACS >0)	Symptom subset with minimum ΔACS (Poor − Good)	Symptom subset with maximum ΔACS (Poor − Good)
3	56	0.22	0.43	0.52	0.61	0.83	100%	Nausea; Sad; Cough	Fatigue; Pain; Shortness of breath
4	70	0.26	0.45	0.52	0.59	0.76	100%	Constipation; Nausea; Sad; Cough	Decreased appetite; Fatigue; Pain; Shortness of breath
5	56	0.33	0.46	0.52	0.58	0.69	100%	Constipation; Decreased appetite; Nausea; Sad; Cough	Constipation; Decreased appetite; Fatigue; Pain; Shortness of breath
6	28	0.39	0.48	0.51	0.56	0.63	100%	Constipation; Decreased appetite; Nausea; Pain; Sad; Cough	Constipation; Decreased appetite; Fatigue; Pain; Sad; Shortness of breath
7	8	0.46	0.49	0.52	0.55	0.57	100%	Constipation; Decreased appetite; Fatigue; Nausea; Pain; Sad; Cough	Constipation; Decreased appetite; Fatigue; Pain; Sad; Shortness of breath; Cough
8	1	0.51	0.51	0.51	0.51	0.51	100%	All 8 symptomatic AEs	All 8 symptomatic AEs

Note. K = Subset size

Table S12. Distribution of differences in mean PRO-CTCAE ACS between EORTC-based physical functioning groups (Poor – Good) across all enumerated symptom subsets by subset size *(k)*, Breast Cancer Cohort

K	Number of enumerated symptom subsets	ΔACS (min)	ΔACS (25th percentile)	ΔACS (median)	ΔACS (75th percentile)	ΔACS (max)	Proportion (ΔACS >0)	Symptom subset with minimum ΔACS (Poor − Good)	Symptom subset with maximum ΔACS (Poor − Good)
3	560	0.26	0.54	0.65	0.76	1.04	100%	Diarrhea; Dizziness; Heart palpitations	Fatigue; Pain; Aching joints
4	1820	0.28	0.56	0.66	0.74	0.98	100%	Diarrhea; Nausea; Dizziness; Heart palpitations	Fatigue; Pain; Taste changes; Aching joints
5	4368	0.32	0.58	0.67	0.74	0.96	100%	Diarrhea; Nausea; Dizziness; Heart palpitations; Memory	Fatigue; Pain; Concentration; Taste changes; Aching joints
6	8008	0.33	0.60	0.67	0.73	0.92	100%	Hair loss; Diarrhea; Nausea; Dizziness; Heart palpitations; Memory	Swelling; Fatigue; Pain; Concentration; Taste changes; Aching joints
7	11440	0.40	0.61	0.67	0.73	0.90	100%	Constipation; Hair loss; Diarrhea; Nausea; Dizziness; Heart palpitations; Memory	Fatigue; Pain; Concentration; Taste changes; Shortness of breath; Aching joints; Memory
8	12870	0.39	0.62	0.67	0.72	0.87	100%	Constipation; Hair loss; Diarrhea; Nausea; Aching joints; Dizziness; Heart palpitations; Memory	Swelling; Fatigue; Insomnia; Pain; Concentration; Taste changes; Shortness of breath; Aching joints
9	11440	0.46	0.63	0.68	0.72	0.86	100%	Constipation; Hair loss; Diarrhea; Nausea; Numbness/Tingling; Aching joints; Dizziness; Heart palpitations; Memory	Swelling; Fatigue; Insomnia; Pain; Concentration; Taste changes; Shortness of breath; Aching joints; Memory
10	8008	0.49	0.64	0.68	0.71	0.83	100%	Swelling; Constipation; Hair loss; Diarrhea; Nausea; Numbness/Tingling; Aching joints; Dizziness; Heart palpitations; Memory	Swelling; Fatigue; Insomnia; Numbness/Tingling; Pain; Concentration; Taste changes; Shortness of breath; Aching joints; Memory
11	4368	0.52	0.64	0.68	0.71	0.81	100%	Constipation; Hair loss; Diarrhea; Nausea; Numbness/Tingling; Concentration; Shortness of breath; Aching joints; Dizziness; Heart palpitations; Memory	Swelling; Fatigue; Insomnia; Numbness/Tingling; Pain; Concentration; Taste changes; Shortness of breath; Aching joints; Dizziness; Memory
12	1820	0.55	0.65	0.68	0.70	0.78	100%	Swelling; Constipation; Hair loss; Insomnia; Diarrhea; Nausea; Numbness/Tingling; Shortness of breath; Aching joints; Dizziness; Heart palpitations; Memory	Swelling; Fatigue; Hair loss; Insomnia; Numbness/Tingling; Pain; Concentration; Taste changes; Shortness of breath; Aching joints; Dizziness; Memory
13	560	0.58	0.66	0.68	0.70	0.76	100%	Swelling; Constipation; Hair loss; Insomnia; Diarrhea; Nausea;	Swelling; Fatigue; Hair loss; Insomnia; Numbness/Tingling; Pain;

								Numbness/Tingling; Concentration; Shortness of breath; Aching joints; Dizziness; Heart palpitations; Memory	Concentration; Taste changes; Shortness of breath; Aching joints; Dizziness; Heart palpitations; Memory
14	120	0.61	0.66	0.68	0.69	0.73	100%	Swelling; Constipation; Hair loss; Insomnia; Diarrhea; Nausea; Numbness/Tingling; Concentration; Taste changes; Shortness of breath; Aching joints; Dizziness; Heart palpitations; Memory	Swelling; Constipation; Fatigue; Hair loss; Insomnia; Numbness/Tingling; Pain; Concentration; Taste changes; Shortness of breath; Aching joints; Dizziness; Heart palpitations; Memory
15	16	0.64	0.67	0.68	0.69	0.71	100%	Swelling; Constipation; Hair loss; Insomnia; Diarrhea; Nausea; Numbness/Tingling; Pain; Concentration; Taste changes; Shortness of breath; Aching joints; Dizziness; Heart palpitations; Memory	Swelling; Constipation; Fatigue; Hair loss; Insomnia; Nausea; Numbness/Tingling; Pain; Concentration; Taste changes; Shortness of breath; Aching joints; Dizziness; Heart palpitations; Memory
16	1	0.68	0.68	0.68	0.68	0.68	100%	All 16 symptomatic AEs	All 16 symptomatic AEs

Note. K = Subset size

Table S13. Distribution of differences in mean PRO-CTCAE ACS between EORTC-based physical functioning groups (Poor – Good) across all enumerated symptom subsets by subset size *(k)*, Head and Neck Cancer Cohort

K	Number of enumerated symptom subsets	ΔACS (min)	ΔACS (25th percentile)	ΔACS (median)	ΔACS (75th percentile)	ΔACS (max)	Proportion (ΔACS >0)	Symptom subset with minimum ΔACS (Poor − Good)	Symptom subset with maximum ΔACS (Poor − Good)
3	680	0.28	0.52	0.59	0.65	0.85	100%	Insomnia; Hoarseness; Radiation skin reaction	Decreased appetite; Fatigue; Cough
4	2380	0.30	0.53	0.59	0.65	0.87	100%	Insomnia; Vomiting; Hoarseness; Radiation skin reaction	Decreased appetite; Fatigue; Cough; Cracking at corners of mouth (cheilosis/cheilitis)
5	6188	0.35	0.54	0.59	0.64	0.80	100%	Insomnia; Mouth/throat sore; Vomiting; Hoarseness; Radiation skin reaction	Decreased appetite; Dry mouth; Fatigue; Cough; Cracking at corners of mouth (cheilosis/cheilitis)
6	12376	0.34	0.55	0.59	0.63	0.81	100%	Insomnia; Mouth/throat sore; Vomiting; Difficulty swallowing; Hoarseness; Radiation skin reaction	Decreased appetite; Dry mouth; Fatigue; Cough; Difficulty swallowing; Cracking at corners of mouth (cheilosis/cheilitis)
7	19448	0.40	0.55	0.59	0.63	0.76	100%	Insomnia; Mouth/throat sore; Nausea; Vomiting; Difficulty swallowing; Hoarseness; Radiation skin reaction	Decreased appetite; Dry mouth; Fatigue; Taste changes; Cough; Difficulty swallowing; Cracking at corners of mouth (cheilosis/cheilitis)
8	24310	0.40	0.56	0.59	0.63	0.75	100%	Insomnia; Mouth/throat sore; Nausea; Vomiting; Difficulty swallowing; Hoarseness; Radiation skin reaction; Cracking at corners of mouth (cheilosis/cheilitis)	Decreased appetite; Dry mouth; Fatigue; Taste changes; Cough; Difficulty swallowing; Radiation skin reaction; Cracking at corners of mouth (cheilosis/cheilitis)
9	24310	0.45	0.56	0.59	0.63	0.73	100%	Constipation; Insomnia; Mouth/throat sore; Nausea; Vomiting; Difficulty swallowing; Hoarseness; Radiation skin reaction; Cracking at corners of mouth (cheilosis/cheilitis)	Decreased appetite; Dry mouth; Fatigue; Pain; Taste changes; Cough; Difficulty swallowing; Radiation skin reaction; Cracking at corners of mouth (cheilosis/cheilitis)
10	19448	0.45	0.56	0.59	0.62	0.71	100%	Anxious; Insomnia; Mouth/throat sore; Nausea; Vomiting; Cough; Difficulty swallowing; Hoarseness; Radiation skin reaction; Cracking at corners of mouth (cheilosis/cheilitis)	Anxious; Constipation; Decreased appetite; Fatigue; Pain; Taste changes; Sad; Cough; Difficulty swallowing; Cracking at corners of mouth (cheilosis/cheilitis)
11	12376	0.48	0.57	0.60	0.62	0.70	100%	Anxious; Constipation; Insomnia; Mouth/throat sore; Nausea; Vomiting; Cough; Difficulty swallowing;	Anxious; Constipation; Decreased appetite; Fatigue; Nausea; Pain; Taste changes; Sad; Cough; Difficulty

								Hoarseness; Radiation skin reaction; Cracking at corners of mouth (cheilosis/cheilitis)	swallowing; Cracking at corners of mouth (cheilosis/cheilitis)
12	6188	0.50	0.57	0.59	0.61	0.69	100%	Anxious; Constipation; Dry mouth; Insomnia; Mouth/throat sore; Nausea; Taste changes; Vomiting; Difficulty swallowing; Hoarseness; Radiation skin reaction; Cracking at corners of mouth (cheilosis/cheilitis)	Anxious; Constipation; Decreased appetite; Dry mouth; Fatigue; Nausea; Pain; Taste changes; Sad; Cough; Difficulty swallowing; Cracking at corners of mouth (cheilosis/cheilitis)
13	2380	0.52	0.58	0.60	0.61	0.68	100%	Anxious; Constipation; Dry mouth; Insomnia; Mouth/throat sore; Nausea; Taste changes; Vomiting; Cough; Difficulty swallowing; Hoarseness; Radiation skin reaction; Cracking at corners of mouth (cheilosis/cheilitis)	Anxious; Constipation; Decreased appetite; Dry mouth; Fatigue; Nausea; Pain; Taste changes; Sad; Cough; Difficulty swallowing; Radiation skin reaction; Cracking at corners of mouth (cheilosis/cheilitis)
14	680	0.53	0.58	0.59	0.61	0.66	100%	Anxious; Constipation; Dry mouth; Insomnia; Mouth/throat sore; Nausea; Pain; Taste changes; Vomiting; Cough; Difficulty swallowing; Hoarseness; Radiation skin reaction; Cracking at corners of mouth (cheilosis/cheilitis)	Anxious; Constipation; Decreased appetite; Dry mouth; Fatigue; Mouth/throat sore; Nausea; Pain; Taste changes; Sad; Cough; Difficulty swallowing; Radiation skin reaction; Cracking at corners of mouth (cheilosis/cheilitis)
15	136	0.55	0.59	0.60	0.61	0.64	100%	Anxious; Constipation; Dry mouth; Insomnia; Mouth/throat sore; Nausea; Pain; Taste changes; Sad; Vomiting; Cough; Difficulty swallowing; Hoarseness; Radiation skin reaction; Cracking at corners of mouth (cheilosis/cheilitis)	Anxious; Constipation; Decreased appetite; Dry mouth; Fatigue; Insomnia; Mouth/throat sore; Nausea; Pain; Taste changes; Sad; Cough; Difficulty swallowing; Radiation skin reaction; Cracking at corners of mouth (cheilosis/cheilitis)
16	17	0.57	0.59	0.59	0.60	0.61	100%	Anxious; Constipation; Decreased appetite; Dry mouth; Insomnia; Mouth/throat sore; Nausea; Pain; Taste changes; Sad; Vomiting; Cough; Difficulty swallowing; Hoarseness; Radiation skin reaction; Cracking at corners of mouth (cheilosis/cheilitis)	Anxious; Constipation; Decreased appetite; Dry mouth; Fatigue; Insomnia; Mouth/throat sore; Nausea; Pain; Taste changes; Sad; Cough; Difficulty swallowing; Hoarseness; Radiation skin reaction; Cracking at corners of mouth (cheilosis/cheilitis)
17	1	0.60	0.60	0.60	0.60	0.60	100%	All 17 symptomatic AEs	All 17 symptomatic AEs

Note. K = Subset size

Table S14. Distribution of differences in mean PRO-CTCAE ACS between ECOG PS groups (ECOG 2-4 – ECOG 0-1) across all enumerated symptom subsets by subset size *(k)*, Lung Cancer Cohort

K	Number of enumerated symptom subsets	ΔACS (min)	ΔACS (25th percentile)	ΔACS (median)	ΔACS (75th percentile)	ΔACS (max)	Proportion (ΔACS >0)	Symptom subset with minimum ΔACS (Poor − Good)	Symptom subset with maximum ΔACS (Poor − Good)
3	56	0.27	0.35	0.38	0.43	0.49	100%	Decreased appetite; Sad; Cough	Constipation; Fatigue; Pain
4	70	0.28	0.35	0.39	0.42	0.48	100%	Decreased appetite; Nausea; Sad; Cough	Constipation; Fatigue; Pain; Shortness of breath
5	56	0.31	0.36	0.39	0.41	0.46	100%	Decreased appetite; Nausea; Pain; Sad; Cough	Constipation; Fatigue; Nausea; Pain; Shortness of breath
6	28	0.33	0.37	0.39	0.40	0.43	100%	Constipation; Decreased appetite; Nausea; Pain; Sad; Cough	Constipation; Fatigue; Nausea; Pain; Sad; Shortness of breath
7	8	0.35	0.37	0.38	0.40	0.40	100%	Constipation; Decreased appetite; Nausea; Pain; Sad; Shortness of breath; Cough	Constipation; Decreased appetite; Fatigue; Nausea; Pain; Sad; Shortness of breath
8	1	0.38	0.38	0.38	0.38	0.38	100%	All 8 symptomatic AEs	All 8 symptomatic AEs

Note. K = Subset size

Table S15. Distribution of differences in mean PRO-CTCAE ACS between ECOG PS groups (ECOG 2-4 – ECOG 0-1) across all enumerated symptom subsets by subset size *(k)*, Breast Cancer Cohort

K	Number of enumerated symptom subsets	ΔACS (min)	ΔACS (25th percentile)	ΔACS (median)	ΔACS (75th percentile)	ΔACS (max)	Proportion (ΔACS >0)	Symptom subset with minimum ΔACS (Poor − Good)	Symptom subset with maximum ΔACS (Poor − Good)
3	560	-0.02	0.36	0.52	0.70	1.52	99.5%	Swelling; Diarrhea; Memory	Constipation; Aching joints; Dizziness
4	1820	-0.01	0.37	0.49	0.61	1.29	99.9%	Diarrhea; Nausea; Heart palpitations; Memory	Constipation; Aching joints; Dizziness; Memory
5	4368	0.07	0.40	0.51	0.61	1.34	100%	Swelling; Hair loss; Diarrhea; Heart palpitations; Memory	Constipation; Hair loss; Aching joints; Dizziness; Memory
6	8008	0.08	0.40	0.49	0.57	1.19	100%	Diarrhea; Nausea; Taste changes; Aching joints; Heart palpitations; Memory	Constipation; Fatigue; Aching joints; Dizziness; Heart palpitations; Memory
7	11440	0.16	0.42	0.49	0.57	1.24	100%	Swelling; Diarrhea; Nausea; Taste changes; Aching joints; Heart palpitations; Memory	Constipation; Fatigue; Hair loss; Aching joints; Dizziness; Heart palpitations; Memory
8	12870	0.18	0.42	0.49	0.55	0.87	100%	Hair loss; Diarrhea; Nausea; Taste changes; Aching joints; Dizziness; Heart palpitations; Memory	Constipation; Fatigue; Pain; Shortness of breath; Aching joints; Dizziness; Heart palpitations; Memory
9	11440	0.22	0.43	0.49	0.55	0.79	100%	Swelling; Hair loss; Insomnia; Diarrhea; Nausea; Taste changes; Aching joints; Heart palpitations; Memory	Constipation; Fatigue; NumbnessTingling; Pain; Shortness of breath; Aching joints; Dizziness; Heart palpitations; Memory
10	8008	0.23	0.43	0.48	0.54	0.73	100%	Swelling; Hair loss; Insomnia; Diarrhea; Nausea; Concentration; Taste changes; Aching joints; Heart palpitations; Memory	Constipation; Fatigue; NumbnessTingling; Pain; Concentration; Shortness of breath; Aching joints; Dizziness; Heart palpitations; Memory
11	4368	0.26	0.45	0.49	0.53	0.68	100%	Swelling; Hair loss; Insomnia; Diarrhea; Nausea; Concentration; Taste changes; Aching joints; Dizziness; Heart palpitations; Memory	Constipation; Fatigue; Insomnia; NumbnessTingling; Pain; Concentration; Shortness of breath; Aching joints; Dizziness; Heart palpitations; Memory
12	1820	0.28	0.45	0.48	0.52	0.64	100%	Swelling; Hair loss; Insomnia; Diarrhea; Nausea; NumbnessTingling; Concentration; Taste changes; Aching joints; Dizziness; Heart palpitations; Memory	Swelling; Constipation; Fatigue; Insomnia; NumbnessTingling; Pain; Concentration; Shortness of breath; Aching joints; Dizziness; Heart palpitations; Memory

13	560	0.33	0.45	0.49	0.52	0.60	100%	Swelling; Constipation; Hair loss; Insomnia; Diarrhea; Nausea; NumbnessTingling; Concentration; Taste changes; Aching joints; Dizziness; Heart palpitations; Memory	Swelling; Constipation; Fatigue; Insomnia; NumbnessTingling; Pain; Concentration; Taste changes; Shortness of breath; Aching joints; Dizziness; Heart palpitations; Memory
14	120	0.37	0.45	0.48	0.51	0.56	100%	Swelling; Constipation; Hair loss; Insomnia; Diarrhea; Nausea; NumbnessTingling; Concentration; Taste changes; Shortness of breath; Aching joints; Dizziness; Heart palpitations; Memory	Swelling; Constipation; Fatigue; Hair loss; Insomnia; NumbnessTingling; Pain; Concentration; Taste changes; Shortness of breath; Aching joints; Dizziness; Heart palpitations; Memory
15	16	0.43	0.47	0.49	0.50	0.53	100%	Swelling; Constipation; Hair loss; Insomnia; Diarrhea; Nausea; NumbnessTingling; Pain; Concentration; Taste changes; Shortness of breath; Aching joints; Dizziness; Heart palpitations; Memory	Swelling; Constipation; Fatigue; Hair loss; Insomnia; Nausea; NumbnessTingling; Pain; Concentration; Taste changes; Shortness of breath; Aching joints; Dizziness; Heart palpitations; Memory
16	1	0.48	0.48	0.48	0.48	0.48	100%	All 16 symptomatic AEs	All 16 symptomatic AEs

Note. K = Subset size

Table S16. Distribution of differences in mean PRO-CTCAE ACS between ECOG PS groups (ECOG 2-4 – ECOG 0-1) across all enumerated symptom subsets by subset size *(k)*, Head and Neck Cancer Cohort

K	Number of enumerated symptom subsets	ΔACS (min)	ΔACS (25th percentile)	ΔACS (median)	ΔACS (75th percentile)	ΔACS (max)	Proportion (ΔACS >0)	Symptom subset with minimum ΔACS (Poor − Good)	Symptom subset with maximum ΔACS (Poor − Good)
3	680	-0.12	0.07	0.13	0.19	0.37	94.7%	Insomnia; Nausea; Difficulty swallowing	Fatigue; Pain; Cough
4	2380	-0.06	0.10	0.15	0.20	0.39	98.6%	Insomnia; Mouth/throat sore; Nausea; Difficulty swallowing	Fatigue; Pain; Cough; Cracking at corners of mouth (cheilosis/cheilitis)
5	6188	-0.05	0.10	0.14	0.19	0.38	99.2%	Insomnia; Mouth/throat sore; Nausea; Taste changes; Difficulty swallowing	Constipation; Fatigue; Pain; Cough; Cracking at corners of mouth (cheilosis/cheilitis)
6	12376	-0.02	0.11	0.15	0.19	0.36	99.9%	Anxious; Insomnia; Mouth/throat sore; Nausea; Taste changes; Difficulty swallowing	Constipation; Fatigue; Pain; Cough; Radiation skin reaction; Cracking at corners of mouth (cheilosis/cheilitis)
7	19448	-0.02	0.11	0.15	0.18	0.34	99.98%	Anxious; Dry mouth; Insomnia; Mouth/throat sore; Nausea; Taste changes; Difficulty swallowing	Constipation; Fatigue; Pain; Sad; Cough; Radiation skin reaction; Cracking at corners of mouth (cheilosis/cheilitis)
8	24310	0.00	0.12	0.15	0.18	0.32	100%	Anxious; Dry mouth; Insomnia; Mouth/throat sore; Nausea; Taste changes; Sad; Difficulty swallowing	Constipation; Fatigue; Pain; Sad; Cough; Hoarseness; Radiation skin reaction; Cracking at corners of mouth (cheilosis/cheilitis)
9	24310	0.02	0.12	0.15	0.18	0.30	100%	Anxious; Dry mouth; Insomnia; Mouth/throat sore; Nausea; Taste changes; Sad; Difficulty swallowing; Hoarseness	Anxious; Constipation; Fatigue; Pain; Sad; Cough; Hoarseness; Radiation skin reaction; Cracking at corners of mouth (cheilosis/cheilitis)
10	19448	0.05	0.13	0.15	0.17	0.28	100%	Anxious; Decreased appetite; Dry mouth; Insomnia; Mouth/throat sore; Nausea; Pain; Taste changes; Sad; Difficulty swallowing	Constipation; Dry mouth; Fatigue; Pain; Sad; Cough; Difficulty swallowing; Hoarseness; Radiation skin reaction; Cracking at corners of mouth (cheilosis/cheilitis)
11	12376	0.06	0.13	0.15	0.17	0.26	100%	Anxious; Decreased appetite; Dry mouth; Insomnia; Mouth/throat sore; Nausea; Pain; Taste changes; Sad; Difficulty swallowing; Hoarseness	Constipation; Decreased appetite; Dry mouth; Fatigue; Pain; Sad; Cough; Difficulty swallowing; Hoarseness; Radiation skin reaction; Cracking at corners of mouth (cheilosis/cheilitis)
12	6188	0.08	0.13	0.15	0.17	0.25	100%	Anxious; Decreased appetite; Dry mouth; Fatigue; Insomnia;	Anxious; Constipation; Decreased appetite; Fatigue; Pain; Sad; Vomiting;

								Mouth/throat sore; Nausea; Pain; Taste changes; Sad; Difficulty swallowing; Hoarseness	Cough; Difficulty swallowing; Hoarseness; Radiation skin reaction; Cracking at corners of mouth (cheilosis/cheilitis)
13	2380	0.09	0.13	0.15	0.17	0.23	100%	Anxious; Constipation; Decreased appetite; Dry mouth; Insomnia; Mouth/throat sore; Nausea; Pain; Taste changes; Sad; Difficulty swallowing; Hoarseness; Radiation skin reaction	Anxious; Constipation; Decreased appetite; Dry mouth; Fatigue; Pain; Sad; Vomiting; Cough; Difficulty swallowing; Hoarseness; Radiation skin reaction; Cracking at corners of mouth (cheilosis/cheilitis)
14	680	0.11	0.14	0.15	0.16	0.21	100%	Anxious; Decreased appetite; Dry mouth; Insomnia; Mouth/throat sore; Nausea; Taste changes; Sad; Vomiting; Cough; Difficulty swallowing; Hoarseness; Radiation skin reaction; Cracking at corners of mouth (cheilosis/cheilitis)	Anxious; Constipation; Decreased appetite; Dry mouth; Fatigue; Mouth/throat sore; Pain; Sad; Vomiting; Cough; Difficulty swallowing; Hoarseness; Radiation skin reaction; Cracking at corners of mouth (cheilosis/cheilitis)
15	136	0.12	0.14	0.15	0.16	0.20	100%	Anxious; Constipation; Decreased appetite; Dry mouth; Fatigue; Insomnia; Mouth/throat sore; Nausea; Pain; Taste changes; Sad; Vomiting; Difficulty swallowing; Hoarseness; Radiation skin reaction	Anxious; Constipation; Decreased appetite; Dry mouth; Fatigue; Mouth/throat sore; Pain; Taste changes; Sad; Vomiting; Cough; Difficulty swallowing; Hoarseness; Radiation skin reaction; Cracking at corners of mouth (cheilosis/cheilitis)
16	17	0.14	0.14	0.15	0.16	0.17	100%	Anxious; Constipation; Decreased appetite; Dry mouth; Insomnia; Mouth/throat sore; Nausea; Pain; Taste changes; Sad; Vomiting; Cough; Difficulty swallowing; Hoarseness; Radiation skin reaction; Cracking at corners of mouth (cheilosis/cheilitis)	Anxious; Constipation; Decreased appetite; Dry mouth; Fatigue; Mouth/throat sore; Nausea; Pain; Taste changes; Sad; Vomiting; Cough; Difficulty swallowing; Hoarseness; Radiation skin reaction; Cracking at corners of mouth (cheilosis/cheilitis)
17	1	0.15	0.15	0.15	0.15	0.15	100%	All 17 symptomatic AEs	All 17 symptomatic AEs

Note. K = Subset size

Table S17. Additional sensitivity analyses examining the impact of sequentially removing terms starting from those symptom terms that were least endorsed by patients until reaching a reduced set of 3 symptom terms, Lung Cancer Cohort

K	Sequentially removed AE term	Test-retest Reliability (GRC-defined stable patients)	Responsiveness					Known-groups Validity (Physical Functioning)				Known-groups Validity (ECOG PS)			
		ICC (95% CI)	*r*	Improved: SRM	No change: SRM	Worsened: SRM	Trend test p-value	*r*	Good Physical Functioning mean ACS	Poor Physical Functioning mean ACS	Cohen's d	*r*	ECOG 0-1 mean ACS	ECOG 2-4 mean ACS	Cohen's d
8	-	0.80 (0.70, 0.87)	0.24	0.30	0.15	-0.37	.001	0.41	0.71	1.23	0.91 (0.59, 1.23)	0.27	0.93	1.31	0.65 (0.29,1.00)
7	Pain	0.78 (0.66, 0.86)	0.24	0.30	0.14	-0.31	.003	0.42	0.69	1.21	0.95 (0.62, 1.28)	0.27	0.91	1.29	0.65 (0.30,1.01)
6	Nausea	0.78 (0.67, 0.86)	0.23	0.28	0.11	-0.28	.002	0.42	0.70	1.23	0.95 (0.62, 1.27)	0.27	0.92	1.32	0.67 (0.31,1.02)
5	Sadness	0.79 (0.67, 0.86)	0.20	0.30	0.12	-0.20	.009	0.46	0.72	1.33	1.04 (0.71, 1.36)	0.26	0.99	1.39	0.64 (0.28,0.99)
4	Constipation	0.81 (0.71, 0.88)	0.17	0.28	0.10	-0.11	.048	0.47	0.69	1.35	1.08 (0.75, 1.41)	0.25	0.99	1.39	0.59 (0.24,0.95)
3	Decreased appetite	0.80 (0.69, 0.87)	0.18	0.28	0.16	-0.13	.032	0.46	0.74	1.44	1.07 (0.74, 1.39)	0.26	1.05	1.50	0.62 (0.26,0.98)

Note. *K*=Number of symptom term sets included in ACS calculation. Sequential term removal was guided by the patients' and health care professionals' relevance scoring published in Veldhuijzen et al. (2021). After removing terms until 3 symptom terms are left, the 3 symptom terms that were left last were fatigue, shortness of breath, and cough.

Table S18. Additional sensitivity analyses examining the impact of sequentially removing terms starting from those symptom terms that were least endorsed by patients until reaching a reduced set of 3 symptom terms, Breast Cancer Cohort

K	Sequentially removed AE term	Test-retest Reliability (GRC-defined stable patients)	Responsiveness					Known-groups Validity (Physical Functioning)				Known-groups Validity (ECOG PS)			
		ICC (95% CI)	r	Improved: SRM	No change: SRM	Worsened: SRM	Trend test p-value	*r*	Good Physical Functioning mean ACS	Poor Physical Functioning mean ACS	Cohen's d	*r*	ECOG 0-1 mean ACS	ECOG 2-4 mean ACS	Cohen's d
16	-	0.84 (0.77, 0.89)	0.29	0.29	0.14	-0.40	<.001	0.61	0.52	1.19	1.59 (1.28,1.89)	0.22	0.74	1.22	0.91 (0.38,1.44)
15	Swelling	0.84 (0.77, 0.89)	0.29	0.28	0.15	-0.42	<.001	0.61	0.55	1.14	1.58 (1.27,1.89)	0.24	0.74	1.24	0.95 (0.42,1.48)
14	Memory	0.83 (0.76, 0.88)	0.28	0.28	0.14	-0.43	<.001	0.61	0.52	1.20	1.57 (1.26,1.88)	0.24	0.74	1.25	0.96 (0.43,1.50)
13	Heart palpitations	0.84 (0.77, 0.89)	0.30	0.28	0.13	-0.45	<.001	0.63	0.51	1.21	1.67 (1.35,1.99)	0.25	0.73	1.26	1.01 (0.48,1.54)
12	Shortness of breath	0.83 (0.75, 0.88)	0.30	0.29	0.14	-0.46	<.001	0.61	0.54	1.23	1.59 (1.28,1.91)	0.23	0.76	1.26	0.93 (0.40,1.46)
11	Dizziness	0.83 (0.76, 0.88)	0.30	0.27	0.13	-0.59	<.001	0.61	0.55	1.26	1.57 (1.26,1.89)	0.22	0.78	1.27	0.88 (0.35,1.41)
10	Taste changes	0.84 (0.78, 0.89)	0.28	0.26	0.13	-0.43	<.001	0.59	0.58	1.28	1.50 (1.19,1.81)	0.22	0.81	1.30	0.87 (0.34,1.40)
9	Constipation	0.84 (0.77, 0.89)	0.29	0.27	0.08	-0.41	<.001	0.59	0.57	1.28	1.50 (1.18,1.81)	0.21	0.80	1.29	0.85 (0.31,1.38)
8	Diarrhea	0.85 (0.79, 0.90)	0.27	0.28	0.03	-0.38	<.001	0.61	0.59	1.38	1.56 (1.25,1.87)	0.22	0.86	1.40	0.88 (0.34,1.41)
7	General Pain	0.83 (0.75, 0.88)	0.27	0.25	0.08	-0.37	<.001	0.58	0.57	1.28	1.47 (1.15,1.78)	0.21	0.79	1.29	0.85 (0.32,1.38)
6	Concentration	0.81 (0.73, 0.87)	0.27	0.26	0.08	-0.37	<.001	0.57	0.60	1.33	1.43 (1.12,1.73)	0.20	0.85	1.34	0.79 (0.26,1.32)
5	Joint pain	0.76 (0.67, 0.83)	0.24	0.25	0.08	-0.39	<.001	0.57	0.59	1.32	1.42 (1.11,1.73)	0.20	0.84	1.31	0.78 (0.25,1.31)
4	Hair loss	0.76 (0.66, 0.83)	0.28	0.30	0.08	-0.43	<.001	0.56	0.59	1.29	1.40 (1.08,1.71)	0.22	0.80	1.32	0.88 (0.33,1.43)

3	Insomnia	0.70 (0.58, 0.79)	0.26	0.30	0.03	-0.40	<.001	0.56	0.58	1.34	1.39 (1.09,1.69)	0.22	0.83	1.40	0.88 (0.34,1.40)

Note. K=Number of symptom term sets included in ACS calculation. Sequential term removal was guided by the ranking score that combined both symptom prevalence and patient-rated importance published in Günther et al. (2023). After removing terms until 3 symptom terms are left, the 3 symptom terms that were left last were fatigue, numbness/tingling, and nausea.

Table S19. Additional sensitivity analyses examining the impact of sequentially removing terms starting from those symptom terms that were least endorsed by patients until reaching a reduced set of 3 symptom terms, Head and Neck Cancer Cohort

K	Sequentially removed AE term	Test-retest Reliability (GRC-defined stable patients)	Responsiveness					Known-groups Validity (Physical Functioning)				Known-groups Validity (ECOG PS)			
		ICC (95% CI)	*r*	Improved: SRM	No change: SRM	Worsened: SRM	Trend test p-value	*r*	Good Physical Functioning mean ACS	Poor Physical Functioning mean ACS	Cohen's d	*r*	ECOG 0-1 mean ACS	ECOG 2-4 mean ACS	Cohen's d
17	-	0.77 (0.63, 0.86)	0.32	-0.01	-0.20	-0.56	.002	0.49	0.69	1.29	1.14 (0.77,1.50)	0.10	0.90	1.06	0.26 (-0.16,0.67)
16	Anxious	0.77 (0.63, 0.86)	0.24	-0.06	-0.23	-0.59	.003	0.48	0.72	1.31	1.10 (0.74,1.47)	0.10	0.90	1.05	0.25 (-0.16,0.67)
15	Constipation	0.77 (0.63, 0.86)	0.28	-0.02	-0.21	-0.66	.001	0.47	0.69	1.28	1.09 (0.72,1.45)	0.09	0.90	1.04	0.23 (-0.18,0.65)
14	Vomiting	0.77 (0.63, 0.86)	0.27	-0.01	-0.22	-0.63	.002	0.47	0.74	1.36	1.08 (0.71,1.44)	0.09	0.95	1.09	0.22 (-0.20,0.63)
13	Cracking at the corners of the mouth	0.76 (0.62, 0.85)	0.27	-0.03	-0.21	-0.65	.003	0.46	0.77	1.38	1.04 (0.67,1.40)	0.08	0.98	1.11	0.19 (-0.22,0.61)
12	Radiation skin reaction	0.77 (0.63, 0.86)	0.28	0.02	-0.13	-0.62	.002	0.45	0.80	1.41	1.02 (0.66,1.38)	0.07	1.02	1.14	0.18 (-0.24,0.59)
11	Sadness	0.75 (0.60, 0.85)	0.28	-0.01	-0.18	-0.63	.003	0.43	0.81	1.42	0.97 (0.61,1.33)	0.07	1.03	1.14	0.16 (-0.25,0.58)
10	Insomnia	0.75 (0.60, 0.85)	0.30	0.02	-0.21	-0.66	.001	0.43	0.81	1.45	0.98 (0.61,1.33)	0.08	1.04	1.18	0.19 (-0.22,0.61)
9	Cough	0.74 (0.58, 0.84)	0.30	0.03	-0.2	-0.68	.001	0.41	0.86	1.48	0.90 (0.55,1.26)	0.06	1.08	1.20	0.16 (-0.26,0.57)
8	Mouth/throat sore	0.73 (0.58, 0.84)	0.31	0.06	-0.21	-0.71	.001	0.42	0.90	1.55	0.93 (0.57,1.29)	0.07	1.13	1.25	0.16 (-0.25,0.58)
7	Nausea	0.72 (0.56, 0.83)	0.29	0.04	-0.26	-0.66	.003	0.41	0.94	1.61	0.91 (0.55,1.26)	0.08	1.17	1.34	0.20 (-0.21,0.62)
6	General pain	0.74 (0.58, 0.84)	0.30	0.09	-0.26	-0.64	.002	0.41	0.96	1.65	0.91 (0.55,1.26)	0.07	1.20	1.34	0.17 (-0.24,0.59)

5	Hoarseness	0.73 (0.57, 0.83)	0.29	0.04	-0.25	-0.65	.005	0.42	0.99	1.71	0.93 (0.57,1.29)	0.05	1.25	1.36	0.13 (-0.28,0.55)
4	Dry mouth	0.69 (0.51, 0.81)	0.26	-0.06	-0.28	-0.62	.002	0.43	0.94	1.69	0.95 (0.59,1.31)	0.06	1.21	1.35	0.15 (-0.26,0.57)
3	Decreased appetite	0.73 (0.57, 0.84)	0.27	-0.08	-0.35	-0.65	.001	0.43	1.00	1.76	0.95 (0.59,1.31)	0.07	1.27	1.42	0.16 (-0.25,0.58)

Note. K=Number of symptom term sets included in ACS calculation. Sequential term removal was guided by the symptom prevalence published in Sander et al. (2018). After removing terms until 3 symptom terms are left, the 3 symptom terms that were left last were difficulty swallowing, taste changes, and fatigue.

Table S20. Distribution of between-group differences in ACS area under the curve (ΔAUC; cabozantinib minus mitoxantrone-prednisone) across all enumerated symptom subsets by subset size *(k)*

K	Number of enumerated symptom subsets	ΔAUC (min)	Δ AUC (25th percentile)	ΔAUC (median)	Δ AUC (75th percentile)	ΔAUC (max)	Symptom subset with minimum ΔACS (cabozantinib - mitoxantrone)	Symptom subset with maximum ΔACS (cabozantinib - mitoxantrone)
3	120	-0.34	0.86	1.38	1.98	3.26	Shortness of breath, Insomnia, Pain	Diarrhea, Nausea, Decreased appetite
4	210	-0.07	0.98	1.44	1.92	2.94	Shortness of breath, Insomnia, Fatigue, Pain	Diarrhea, Nausea, Decreased appetite, Numbness/tingling (hands/feet)
5	252	0.24	1.11	1.46	1.79	2.71	Constipation, Shortness of breath, Insomnia, Fatigue, Pain	Diarrhea, Nausea, Vomiting, Decreased appetite, Numbness/tingling (hands/feet)
6	210	0.54	1.19	1.46	1.74	2.53	Constipation, Shortness of breath, Numbness/tingling (hands/feet), Insomnia, Fatigue, Pain	Diarrhea, Constipation, Nausea, Vomiting, Decreased appetite, Numbness/tingling (hands/feet)
7	120	0.76	1.23	1.48	1.70	2.19	Constipation, Nausea, Shortness of breath, Numbness/tingling (hands/feet), Insomnia, Fatigue, Pain	Diarrhea, Constipation, Nausea, Vomiting, Decreased appetite, Numbness/tingling (hands/feet), Fatigue
8	45	0.92	1.31	1.51	1.58	1.91	Constipation, Nausea, Vomiting, Shortness of breath, Numbness/tingling (hands/feet), Insomnia, Fatigue, Pain	Diarrhea, Constipation, Nausea, Vomiting, Decreased appetite, Shortness of breath, Numbness/tingling (hands/feet), Fatigue
9	10	1.14	1.40	1.41	1.59	1.69	Constipation, Nausea, Vomiting, Decreased appetite, Shortness of breath, Numbness/tingling (hands/feet), Insomnia, Fatigue, Pain	Diarrhea, Constipation, Nausea, Vomiting, Decreased appetite, Shortness of breath, Numbness/tingling (hands/feet), Insomnia, Fatigue
10	1	1.45	1.45	1.45	1.45	1.45	All 10 symptomatic AEs	All 10 symptomatic AEs

Note. K = Subset size
